# Bibenzyl Derivatives from *Radula voluta* (An Ecuadorian Liverwort): Bioprospecting for Antiprotozoal Properties

**DOI:** 10.3390/molecules30234543

**Published:** 2025-11-25

**Authors:** José Miguel Andrade, Carlos J. Bethencourt-Estrella, Javier Chao-Pellicer, Luis Cartuche, Vladimir Morocho, Ángel Benítez, Rubén L. Rodríguez-Expósito, José E. Piñero, Jacob Lorenzo-Morales, Ana R. Díaz-Marrero, José J. Fernandez

**Affiliations:** 1 Departamento de Química, Universidad Técnica Particular de Loja (UTPL), Calle Paris s/n y Praga, Loja 110107, Ecuador; jmandrade@utpl.edu.eclecartuche@utpl.edu.ec (L.C.); svmorocho@utpl.edu.ec (V.M.); 2 Instituto Universitario de Bio-Orgánica Antonio González (IUBO AG), Universidad de La Laguna (ULL), 38206 La Laguna, Spain; jjfercas@ull.edu.es; 3 Instituto Universitario de Enfermedades Tropicales y Salud Pública de Canarias, Universidad de La Laguna (ULL), Avda. Astrofísico Fco. Sánchez, S/N, 38203 La Laguna, Spain; cbethene@ull.edu.es (C.J.B.-E.); jchaopel@ull.edu.es (J.C.-P.); rrodrige@ull.edu.es (R.L.R.-E.); jpinero@ull.edu.es (J.E.P.); 4 Departamento de Obstetricia y Ginecología, Pediatría, Medicina Preventiva y Salud Pública, Toxicología Medicina Legal y Forense y Parasitología, Universidad de La Laguna (ULL), 38200 La Laguna, Spain; 5 Centro de Investigación Biomédica en Red de Enfermedades Infecciosas (CIBERINFEC), Instituto de Salud Carlos III, 28220 Madrid, Spain; 6 Biodiversidad de Ecosistemas Tropicales-BIETROP, Herbario HUTPL, Departamento de Ciencias Biológicas y Agropecuarias, Universidad Técnica Particular de Loja, San Cayetano s/n, Loja 1101608, Ecuador; arbenitez@utpl.edu.ec; 7 Instituto de Productos Naturales y Agrobiología (IPNA), Consejo Superior de Investigaciones Científicas (CSIC), Avenida Astrofísico Francisco Sánchez 3, 38206 La Laguna, Spain; 8 Biotecnología Marina, IUBO-ULL, Unidad Asociada al IPNA-CSIC, 38206 La Laguna, Spain

**Keywords:** *Radula voluta*, bibenzyls, antiprotozoal activity, *Trypanosoma cruzi*, *Leishmania* spp., *Naegleria fowleri*, *Acanthamoeba castellanii*

## Abstract

Phytochemical investigation of *Radula voluta*, a liverwort species collected in the Ecuadorian Amazon, led to the isolation of four known bibenzyl derivatives: 2-prenyl-3,5-dihydroxy-bibenzyl (**1**), 2-geranyl-3,5-dihydroxybibenzyl (**2**), 2,2-dimethyl-5-phenethyl-2H-chromen-7-ol (**3**), and radulanin L (**4**). Structural elucidation was achieved through extensive NMR and MS analyses, supported by comparison with previously reported data. Compounds **1** and **4** are reported for the first time in *R. voluta*. The crude extract and isolated compounds were evaluated for their in vitro antiprotozoal activity against *Trypanosoma cruzi*, *Leishmania amazonensis*, *Leishmania donovani*, *Naegleria fowleri*, and *Acanthamoeba castellanii* Neff. Among the isolated compounds, bibenzyls **2** and **4** exhibited the most potent activity across multiple protozoan strains. Cytotoxicity was assessed against murine macrophages (J774A.1), obtaining moderate–low toxicities against compounds **1** and **3**. These findings highlight the pharmacological value of liverwort-derived bibenzyls and support further research on *R. voluta* as a promising source of antiparasitic leads.

## 1. Introduction

Infectious diseases remain one of the leading causes of morbidity and mortality across the Americas, with a significant burden concentrated in South America [1]. Many parasitic infections either lack effective therapies or have current treatments that are highly toxic. Among these pathogens, kinetoplastids (such as *Trypanosoma* spp. and *Leishmania* spp.) and free-living amoebae, including *Acanthamoeba* spp. and the highly lethal *Naegleria fowleri*, represent urgent medical challenges. Human infections caused by these protozoa often lead to severe or fatal outcomes, and therapeutic options remain scarce, highlighting the critical need for new bioactive compounds with antiparasitic activity [2,3,4].

Ecuador, located in northwestern South America in the heart of the northern Andes, harbors exceptional botanical bryophyte diversity, with more than 720 liverwort species recorded [5,6]. Liverworts, in particular, represent a fascinating yet underexplored group of plants capable of producing a wide variety of biologically active metabolites. Despite their potential, fewer than 3% of liverwort species have been investigated for their chemical and pharmacological properties. These plants have already demonstrated antiparasitic potential: for example, marchantin A from *Marchantia polymorpha* L. exhibits inhibitory effects in *Plasmodium falciparum*, *Trypanosoma brucei*, *Trypanosoma cruzi*, and *Leishmania donovani* [7], while the essential oil of *Plagiochila porelloides* displays moderate activity against *Trypanosoma brucei* [8]. Despite these promising findings, the chemical composition of essential oils from Ecuadorian liverworts remains poorly studied [9,10,11,12].

Species of the genus *Radula* are leafy liverworts distributed worldwide, recognized for their structural diversity and production of unusual chemical scaffolds. They are particularly rich in aromatic derivatives such as bibenzyls, prenyl bibenzyls, and bis-bibenzyls, which are relatively rare in the plant kingdom [13]. These compounds often contain prenyl or geranyl chains that undergo intramolecular oxidations and cyclizations [14,15,16,17,18], giving rise to unique structures with diverse biological activities, ranging from antimicrobial and antifungal [19], antiviral [20], enzyme-inhibitory [21], vasopressin-inhibitory [22], antioxidant and nitric oxide-inhibitory [14], insect antifeedant [23], antitrypanosomal [24], antithrombin [25], and cytotoxicity [24].

**Figure 1:**
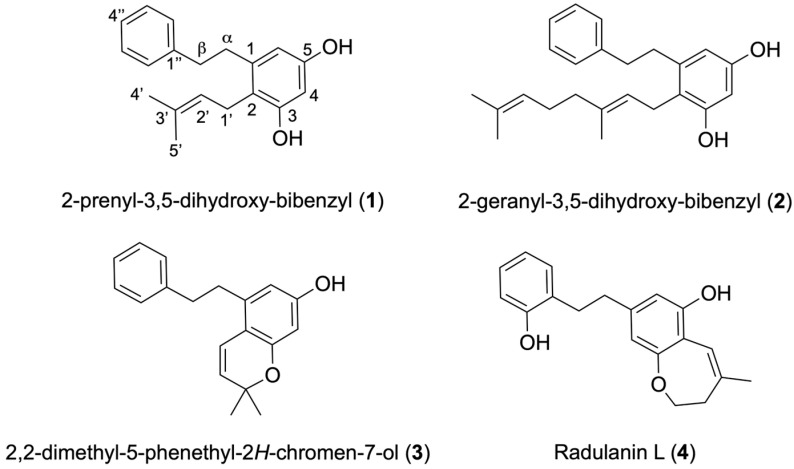
Structure of compounds (**1**–**4**) isolated from *Radula voluta.*

*Radula voluta* is a liverwort that grows abundantly in montane forests and Ecuador páramos [5] between 450 and 4000 m, distributed across provinces such as Azuay, Carchi, Cotopaxi, Chimborazo, Galápagos, Loja, Morona Santiago, Napo, Pastaza, Pichincha, Sucumbíos, Tungurahua, and Zamora Chinchipe [6]. Previous phytochemical studies of this species carried out in Ecuador reported two known bibenzyls, 2-prenyl-3,5-dihydroxy-bibenzyl (**1**) and 2-geranyl-3,5-dihydroxy-bibenzyl (**2**) (Figure 1) [26]. Another chemical study of *R. voluta* carried out in Argentina reported the same compounds isolated in Ecuador [27]. With the aim of exploring the antiparasitic properties of endemic species of Ecuador, this paper reports on the isolation and characterization of four bibenzyl compounds, labeled **1–4**, from *R. voluta* collected in the Ecuadorian Amazon and their biological activity against kinetoplastid parasites and free-living amoeba, highly pathogenic organisms that continue to challenge human health due to the lack of safe and effective treatments.

## 2. Results

### 2.1. Isolation and Characterization

The ethanol extract of *R. voluta* was initially chromatographed on Sephadex LH-20, followed by purification on silica gel, which yielded four compounds (Figure 1). All molecules were characterized by spectroscopic techniques such as GC/MS, 1D and 2D NMR experiments, and further comparison with data from the literature.

The molecular formula of compound **1** was confirmed to be C_19_H_22_O_2_ by HR-ESIMS data, with a peak at *m/z* 281.1534 [M-H]¯ (calc. for C_19_H_21_O_2_ 281.1542). The presence of a non-substituted benzyl group was confirmed by ^1^H NMR signals at [δ 7.29 (2H, m, H-3″-5″), 7.19 (3H, m, H-2″-4″-6″)]. In addition, the ^1^H NMR spectrum of compound **1** contained the signals of a 2,2-dimethylallyl group [δ 1.80 (3H, s, H-5′), 1.73 (3H, s, H-4′), 5.10 (1H, t, *J*= 6.0 Hz, H-2′), 3.30 (2H, d, *J* = 6.8 Hz, H-1′)], two *meta* coupled protons [δ 6.28 (1H, d, *J* = 2.6 Hz, H-6), 6.25 (1H, d, *J* = 2.6 Hz, H-4)] and two benzylic methylene [δ 2.84 (4H, s, H-α-β)]. Based on the above results and corroborated with the literature, the structure of **1** was elucidated to be 2-prenyl-3,5 dihydroxy-bibenzyl [28,29].

Compound **2** had been previously isolated from *R. voluta* [21,22] and other genus species of *Radula* [23,24]. The NMR spectroscopic data of compound **2** led to the assumption of a 2-geranyl-3,5-dihydroxy-bibenzyl, which was supported by data in the literature [17]. Its molecular formula was determined by HR-ESIMS to be *m/z* 349.2175 [M-H]¯ (calc. for C_24_H_29_O_2_ 349.2168).

The structure of 2,2-dimethyl-5-phenethyl-2H-chromen-7-ol (**3**) was established by comparison of its spectral data with those bibenzyls previously reported in liverworts [12,23]. A peak at *m/z* 279.1387 [M-H]¯ (calc. for C_19_H_19_O_2_ 279.1385) in the HR-ESIMS established the molecular formula. This structural transformation was corroborated by ^1^H NMR (δ 7.28, 2H, t; 7.18, 3H, m; 6.19, 2H, d; 2.84, 4H, br s) and by the ^13^C NMR spectrum. This is the first report of the presence of compound **3** in *R. voluta*.

The molecular formula of compound **4** was determined by HR-ESIMS to be *m/z* 295.13399 [M-H]¯ (calc. for C_19_H_19_O_2_ 295.13397). The ^1^H NMR data displayed signals for a disubstituted aromatic ring [δ 7.08 (2H, m, H-4″-6″), 6.86 (1H, ddd, *J* = 7.5, 7.4, 1.2 Hz, H-5″), 6.75 (1H, dd, *J* = 8.4, 1.2 Hz, H-3″)], and signals for a tetrasubstituted aromatic ring [δ 6.55 (1H, d, *J* = 1.6 Hz, H-9), 6.39 (1H, d, *J* = 1.6 Hz, H-7)]. In addition, an olefinic methyl [δ 1.54 (3H, s, H-1′)] and two methylene group were observed [δ 4.40 (2H, br s, H-2), 3.40 (2H, d, *J* = 4.0 Hz, H-5)], which confirmed compound **4** as radulanin L, previously isolated from the following *Radula* species: *R. complanate*, *R. appressa*, *R. constricta*, and *R. tokiensis* [9]. This is the first report of the presence of compound **4** in *R. voluta*.

### 2.2. Antiprotozoal Effect and Cytotoxicity

The isolated compounds were evaluated for their in vitro antiprotozoal activity against the kinetoplastids *Trypanosoma cruzi*, *Leishmania amazonensis*, and *Leishmania donovani*, and the free-living amoebae *Naegleria fowleri* and *Acanthamoeba castellanii* Neff. Table 1 presents the results of the screening for each compound against all tested protozoa. Compounds **2** and **4** show promising antiparasitic activity, since they appear to be active against a broad range of parasites. In contrast, compounds **1** and **3** appear to be the compounds with the lowest activity against most parasites.

As shown in Table 1, *Naegleria fowleri* was the most sensitive protozoan strain, being affected by all the tested compounds. This suggests a potential general mechanism of action for the bibenzyl derivatives, as the parasites show sensitivity at 25 µg/mL. Despite the notable structural differences between compounds (**2**) and (**4**), *Leishmania amazonensis* and *Trypanosoma cruzi* were affected at the same dose level, 25 µg/mL. In contrast, *Leishmania donovani*, and *Acanthamoeba castellanii* were the least affected by the treatments, emerging as the most resistant strains. These findings indicate that the activity of bibenzyl derivatives cannot be clearly differentiated between kinetoplastids and free-living amoebae.

To assess the safety and therapeutic potential of the isolated bibenzyl derivatives, cytotoxicity was evaluated against murine macrophages (J774A.1) using the alamarBlue™ assay. The 50% cytotoxic concentration (CC_50_) effects are summarized in Table 2. Derivative **2** was the most toxic (CC_50_ = 14.32 µg/mL), followed by compound **4** (CC_50_ = 18.18 µg/mL). In contrast, compounds **1** and **3** were the least toxic, with CC_50_ values exceeding 50 µg/mL.

The reference drugs employed in this study are reported in Table 3. Miltefosine exhibited consistent activity against *Leishmania* genus parasites and moderate cytotoxicity. Benznidazole showed activity only against *T*. *cruzi*, while exhibiting low cytotoxicity. In contrast, chlorhexidine and voriconazole were highly effective against *A. castellanii* Neff, with moderate toxicities, even lower than those observed for the bibenzyl derivatives. In the case of *N. fowleri*, the standard treatment, amphotericin B, exhibits low toxicity and high efficacy.

## 3. Discussion

The present study reports for the first time the antiprotozoal activity and cytotoxicity profiles of four bibenzyl derivatives isolated from *Radula voluta*, a liverwort species from the Ecuadorian Amazon. Among these, two compounds, **3** and **4**, are newly reported for this species, expanding the chemotaxonomic knowledge of the genus *Radula*. The biological evaluation targeted a broad spectrum of pathogenic protozoa, including kinetoplastids (*T. cruzi*, *L. amazonensis*, *L. donovani*) and free-living amoebae (*N. fowleri*, *A. castellanii*), which are recognized for their clinical relevance and limited therapeutic options [33,34,35].

Bibenzyls **2** and **4** demonstrated the highest antiprotozoal activity, particularly against *N. fowleri, T. cruzi*, and *L. amazonensis*, with sensibility at 25 µg/mL. These values are comparable to or better than other liverwort-derived secondary metabolites previously reported [36,37,38,39]. However, the cytotoxicity profile revealed that compounds **2** and **4** had low CC_50_ values (14.32 and 18.18 µg/mL). Although some compounds exhibited biological activity, their very low toxicity suggests limited efficacy and renders them unsuitable as potential therapeutic agents, since they could lead to undesirable treatment effects. In contrast, compounds **1** and **3** displayed moderate toxicity (50.11 and 57.85 µg/mL) relative to the reference drugs, indicating a more favorable therapeutic window. Given their comparable activity but lower toxicity in mammalian cells, these compounds appear to pose a reduced risk of adverse effects during treatment.

Given that current treatments against these protozoa are not fully effective and often cause significant side effects, coupled with the limited treatment options, especially for *N. fowleri* [40], compounds demonstrating some activity against these parasites provide a strong impetus for the development of future therapies. This includes both the search for new bibenzyl derivatives that may improve upon those obtained in this study and the generation of novel semi-synthetic derivatives.

Structurally, the presence of the geranyl side chains on the bibenzyl core appears to enhance biological activity in compound **2** compared with the prenylated derivative **1**. This effect is also evident in the toxicity values, with compound **2** showing the highest toxicity rates. Prenylation (or geranylation) of aromatic scaffolds such as the bibenzyl core has been repeatedly associated with enhanced biological activity due to increased lipophilicity and membrane-permeation potential. Therefore, lipophilicity was analyzed using the SwissADME platform [41,42]., which allowed us to predict the ADME parameters, pharmacokinetic properties, drug-like nature, and medicinal chemistry friendliness of compounds **1–4** (S21–S25, Supplementary Material) [41]. The calculated average *Log P* (*n*-octanol/water) value for 2-prenyl-3,5-dihydroxy-bibenzyl (1) (*log P* = 4.36) was significantly lower than that of 2-geranyl-3,5-dihydroxy-bibenzyl (**2**) (*log P* = 5.82), with compound **2** showing the best performance in the predictive model in terms of high passive absorption through the gastrointestinal tract (S25, Supplementary Material). This is consistent with previous reports on prenylated bibenzyls and other phenolic metabolites from liverworts, which have shown improved membrane permeability and biological interaction [43,44]. In contrast, radulanin L (**4**) showed lower values of lipophilicity (*log P* = 3.66) compared with **3** (*log P* = 4.11). The enhanced bioactivity of radulanin L (**4**) suggests that the additional hydroxyl group at position 2″ may play a role in improving its activity. This modification also confers favorable properties for crossing the blood–brain barrier (BBB), as indicated by the predictive model (S25, Supplementary Material).

Overall, the data support the antiprotozoal potential of bibenzyls derived from *Radula voluta*, with compounds **2** and **4** identified as the most effective candidates against all parasites. In contrast, compounds **1** and **3** exhibit the lowest cytotoxicity. These findings support the need for further investigation of the minor components of *Radula voluta* and structural optimization to improve their potential as antiparasitic leads, with future studies focusing on structure–activity relationship (SAR) modeling and semi-synthetic modification to enhance potency and selectivity.

## 4. Materials and Methods

### 4.1. General Information

NMR spectra were acquired on a Bruker AVANCE 500 MHz or 600 MHz (Bruker Biospin, Falländen, Switzerland) instrument spectrometer at 300 K when required. The Bruker AVANCE 600 MHz spectrometer was equipped with a 5 mm TCI inverse detection cryoprobe (Bruker Biospin, Falländen, Switzerland). Standard Bruker NMR pulse sequences were utilized. NMR spectra were obtained by dissolving samples in CDCl_3_ (99.9%). Electrospray ESI high-resolution mass spectra (HRMS) were recorded on a Waters VG-Micromass, model Zab 2F (Waters, Manchester, UK), and a Vion IMS Q-TOF (Waters, Manchester, UK). IR spectra were recorded between 450 and 4000 cm^−1^ on an Agilent Cary 630 FTIR Spectrometer (Agilent Technologies Inc., Santa Clara, CA, USA). The GC-MS analyses were performed on an Agilent Technologies (Wilmington, DE, USA) 6890 N gas chromatograph coupled to an Agilent Technologies 5973 N mass spectrometer detector. Silica gel 60 (Merck KGaA, Darmstadt, Germany, from 0.063 to 0.200 mm) was used as stationary phase for column chromatography. Normal-phase Thin Layer Chromatography (TLC) plates, with fluorescence indicator at 254 nm, were purchased from Sigma-Aldrich. After exposure to UV light (254 and 366 nm), the plates were revealed with a mixture of sulfuric acid and vanillin. The organic solvents, used for CC and TLC, were purchased from Brenntag (Guayaquil, Ecuador) and carefully distilled before using. Reference commercial drugs were used as positive controls in both cytotoxicity and protozoan assays, including miltefosine (Cayman Chemical, Vitro SA, Madrid, Spain), benznidazole (Sigma-Aldrich, Madrid, Spain), chlorhexidine (chlorhexidine digluconate; Alfa Aesar), and voriconazole (Fluka; Sigma-Aldrich Chemistry Ltd., Madrid, Spain). The alamarBlue^TM^ reagent (Life Technologies, Madrid, Spain) was employed as a metabolic indicator.

### 4.2. Plant Material

The samples of liverwort *Radula voluta* were collected in Morona Santiago province, Ecuador, Canton Morona, Sevilla Don Bosco Parish in May 2019, at 2500 m a.s.l. (coordinates: 2°39′37.2″ S., 77°42′39.4″ W). The sample collection was conducted in keeping with Ecuadorian law and authorized by the Ministry of Environment, Water, and Ecological Transition of Ecuador (MAATE) under permit code MAATE-DBI-CM-2022-0248. A voucher specimen (HUTPL AB-1338) has been deposited at the Herbarium HUTPL of the Universidad Técnica Particular de Loja. The identity of the plant material was confirmed by the curator of lichens and bryophytes at the mentioned herbarium. Plants samples that were free of impurities were placed in a dehydrator apparatus at 30 °C for 48 h before extraction.

### 4.3. Extraction and Isolation

Cleaned, air-dried material (200.1 g) was extracted with EtOH at room temperature for 8 days. Following this, ethanol extract was filtered, and the solvent evaporated under vacuum to give a green oil (13.1 g). The crude extract was chromatographed on a Sephadex LH-20 with MeOH (100%) in order remove impurities (chlorophyll) from the compounds of interest. A total of 5 fractions (RvF1–RvF5) were collected.

The RvFr3 (96.68 mg) was subjected to column chromatography on silica gel and eluted with a step gradient solvent system of *n*-hexane/EtOAc (90:10, 80:20, 70:30, *v/v*). Fractions with similar behavior on TLC were combined and evaporated, yielding 7 sub-fractions (RvF3-1 to RvF3-7). The sub-fraction RvF3-2 yielded the compound 2,2-dimethyl-7-hydroxy-5-(2-phenylethyl) chromene (**3**) (11.6 mg). The sub-fraction RvF3-4 yielded the compound 2-geranyl-3,5-dihydroxy bibenzyl (**2**) (10.9 mg). The fraction RvF3-5 contained the compound 3,5 dihydroxy-2-(3-methyl-2-butenyl) bibenzyl (**1**) (31.2 mg).

The fraction RvFr4 (31.3 mg) was submitted to silica column chromatography. The column was eluted with an isocratic system of *n*-hexane/EtOAc (70:30, *v/v*) to obtain four sub-fractions (RvF4-1 to RvF4-4). The RvF4-1 (3.0 mg) was purified by column chromatography on silica gel and eluted with an isocratic system of *n*-hexane/EtOAc (70:30, *v/v*) to obtain three sub-fractions (RvF4-1-1 to RvF4-1-3). The pure substance (RvF4-1-2) obtained in this purification was identified as radulanin L (**4**) (2.2 mg).

#### 4.3.1. 3,5-Dihydroxy-2-(3-methyl-2-butenyl) Bibenzyl (**1**)

Compound (**1**) was obtained as a brown oil, FT-IR (ν, cm_¯_^1^) 3381, 2923, 1602, 1453, 1135, 699; ^1^H NMR (500 MHz, CDCl_3_) δ 7.29 (2H, m, H-3″-5″), 7.19 (3H, m, H-2″-4″-6″), 6.28 (1H, d, *J* = 2.6 Hz, H-6), 6.25 (1H, d, *J* = 2.6 Hz, H-4), 5.39 (1H, br s, OH), 5.18 (1H, br s, OH), 5.10 (1H, t, *J*= 6.0 Hz, H-2′) 3.30 (2H, d, *J* = 6.8 Hz, H-1′), 2.84 (4H, s, H-α-β), 1.80 (3H, s, H-5′), 1.73 (3H, s, H-4′); ^13^C NMR (126 MHz, CDCl_3_) δ 156.03 (C-3), 154.81(C-5), 142.51 (C-1″), 142.10 (C-1), 134.43 (C-3′) 128.78 (C-2″-3″-5″-6″), 126.38 (C-4″), 123.05 (C-2′), 118.02 (C-2), 109.27 (C-6), 101.80 (C-4), 37.92 (C-β), 36.00 (C-α), 26.11 (C-4′), 25.26 (C-1′), 18.32 (C-5′); HR-ESIMS *m/z* 281.1534 [M-H]¯ (calc. for C_19_H_21_O_2_ 281.1542). One- and two-dimensional NMR spectra are included in the Supplementary Material.

#### 4.3.2. 2-Geranyl-3,5-dihydroxy-bibenzyl (**2**)

Compound (**2**) was isolated as a brown oil, FT-IR (ν, cm_¯_^1^) 3381, 2924, 2856, 1597, 1456, 1134, 699; ^1^H NMR (600 MHz, CDCl_3_): δ 7.29 (2H, t, *J* = 7.5 Hz, H-3″-5″), 7.18 (3H, m, H-2″-4″-6″), 6.27 (1H, d, *J* = 2.2 Hz, H-6), 6.25 (1H, d, *J* = 2.8 Hz, H-4), 5.34 (1H, br s, OH), 5.11 (1H, t, *J* = 6.0 Hz, H-2′) 5.04 (1H, t, *J* = 6.6 Hz, H-6′), 3.29 (2H, d, *J* = 6.4 Hz, H-1′), 2.84 (4H, br s, H-α-β), 2.09 (2H, m, H-5′), 2.03 (2H, m, H-4′), 1.79 (3H, s, H-10′), 1.72 (3H, s, H-9′), 1.58 (3H, s, H-8′); ^13^C NMR (151 MHz, CDCl_3_): δ 155.88 (C-3), 154.66 (C-5), 142.22 (C-1), 141.86 (C-1″), 138.10 (C-3′), 134.20 (C-7′), 128.54 (C-2″-3″-5″-6″), 126.14 (C-4″), 123.95 (C-6′), 122.81 (C-2′), 117.70 (C-2), 108.98 (C-6), 101.54 (C-4), 39.78 (C-4′), 37.70 (C-β), 35.78 (C-α), 26.57 (C-5′), 25.87 (C-9′), 25.03 (C-1′), 18.08 (C-10′), 16.39 (C-8′). HR-ESIMS *m/z* 349.2175 [M-H]¯ (calc. for C_24_H_29_O_2_ 349.2168). One- and two-dimensional NMR spectra are included in the Supplementary Material.

#### 4.3.3. 2,2-Dimethyl-5-phenethyl-2H-chromen-7-ol (**3**)

Compound (**3**) yielded a dark-brown oil, FT-IR (ν, cm_¯_^1^) 2921, 2851, 1609, 1456, 1134, 699; ^1^H NMR (600 MHz, CDCl_3_): δ 7.28 (2H, m, H-3″-5″), 7.18 (1H, m, H-4″), 7.18 (2H, m, H-2″-6″), 6.44 (1H, d, *J* = 10.0 Hz, H-4), 6.19 (2H, d, *J* = 2.5 Hz, H-6-8), 5.50 (1H, d, *J* = 9.9 Hz, H-3), 2.84 (4H, br s, H-α-β), 1.40 (6H, s, H-1′-2′). ^13^C NMR (151 MHz, CDCl_3_) δ 156.09 (C-7), 154.78 (C-9), 141.71 (C-1″), 139.24 (C-5), 128.54 (C-2″-3″-5″-6″), 128.10 (C-3), 126.16 (C-4″), 118.87 (C-4), 113.06 (C-10), 108.81 (C-6), 102.15 (C-8), 75.77 (C-2), 37.54 (C-β), 34.53 (C-α), 27.84 (C-1′-2′). HR-ESIMS *m/z* 279.1387 [M-H]¯ (calc. for C_19_H_19_O_2_ 279.1385). One- and two-dimensional NMR spectra are included in the Supplementary Material.

#### 4.3.4. Radulanin L (**4**)

Compound (**4**) was recovered as a light-brown oily residue, FT-IR (ν, cm_¯_^1^) 2923, 2854, 1734, 1457; ^1^H NMR (500 MHz, CDCl_3_) δ 7.08 (2H, m, H-4″-6″), 6.86 (1H, ddd, *J* = 7.5, 7.4, 1.2 Hz, H-5″), 6.75 (1H, dd, *J* = 8.4, 1.2 Hz, H-3″), 6.55 (1H, d, *J* = 1.6 Hz, H-9), 6.39 (1H, d, *J* = 1.6 Hz, H-7), 5.61 (1H, t, *J* = 5.5 Hz, H-4), 4.87 (1H, br s, OH), 4.80 (1H, br s, OH), 4.40 (2H, br s, H-2), 3.40 (2H, d, *J* = 4.0 Hz, H-5), 2.83 (4H, m, H-α-β), 1.54 (3H, s, H-1′). ^13^C NMR (126 MHz, CDCl_3_) δ 159.85 (C-10), 153.64 (C-2″), 152.26 (C-6), 141.76 (C-8), 134.13 (C-3), 130.46 (C-6″), 127.5 (C-1″-4″), 121.03 (C-4-11), 120.85 (C-5″), 115.54 (C-3″), 113.91 (C-9), 111.70 (C-7), 74.41 (C-2), 35.84 (C-α), 32.15 (C-β), 21.84 (C-5), 20.24 (C-1′). HR-ESIMS *m/z* 295.13399 [M-H]¯ (calc. for C_19_H_19_O_2_ 295.13397). One and two-dimensional NMR spectra are included in the Supplementary Material.

### 4.4. Cultures

To carry out the leishmanicidal and trypanocidal screening assays, promastigote forms of *L. amazonensis* (MHOM/BR/77/LTB0016) and *L. donovani* (MHOM/IN/90/GE1F8R) were grown at 26 °C in Schneider’s medium (SND, Sigma-Aldrich, Darmstadt, Germany), supplemented with 10% foetal bovine serum (FBS, VWR, Biowest, Nuaillé, France). Epimastigote forms of *T. cruzi* (Y strain) were cultured at 26 °C in Liver Infusion Tryptose (LIT) medium supplemented with 10% FBS. Trophozoites of *Acanthamoeba castellanii* Neff, genotype T4 (ATCC 30010), and *N. fowleri* (ATCC30808) were grown in Peptone yeast glucose (PYG) medium and Bactocasitone medium supplemented with 10% FBS, respectively. Murine macrophages from cell line J774A.1 (ATCC TIB-67) were cultured at 37 °C in a 5% CO_2_ atmosphere in Dulbecco’s Modified Eagle Medium (DMEM) supplemented with 10% FBS. To develop the assays, the medium of the macrophage cells was changed to RPMI 1640 medium (Gibco, New York, MA, USA).

### 4.5. Preliminary Antiprotozoal Activity

#### 4.5.1. In Vitro Activity Against Epimastigote of *Trypanosoma cruzi* and Promastigote of *Leishmania* spp

In 96-well plates, two concentration dilutions of each compound (25 and 50 µg/mL) were added with parasites at a concentration of 10^6^ cells/mL in a final volume of 200 µL of LIT medium for *T. cruzi* and Schneider for *Leishmania* spp.

After 72 h of incubation at 26 °C, each plate was observed under a microscope to determine the concentration of compounds that allowed the parasites to survive. Observation of morphological changes, decreased parasite motility, or a reduction in parasite numbers indicated that the compound had activity against parasites [45].

#### 4.5.2. In Vitro Activity Against Trophozoites of *Acanthamoeba castellanii* Neff and *Naegleria fowleri*

In 96-well plates, 50 µL of parasites were added to each plate (2.5 × 10^5^ cells/mL of *N. fowleri* in Bactocasitone medium or 5 × 10^4^ cells/mL of *A. castellanii* Neff in PYG medium). After trophozoite attachment, 50 μL of the dilutions (25 and 50 µg/mL) of each molecule were added to each well. After incubation (for 96 h at 26 °C for *A. castellanii* Neff. and 48 h at 37 °C for *N. fowleri*), the wells were examined, as in the kinetoplastid assay, to determine the potential activity of the compounds [46,47].

#### 4.5.3. Results of the Screening

The results against each concentration were represented as (+) when compounds presented activity and the parasite was dead, or (−) when the compounds were not active and the parasites were alive. Each experiment was conducted in triplicate to ensure the result and its repeatability.

### 4.6. Cytotoxicity Assay

The cytotoxicity of the active compounds was assessed against murine macrophage cell line J774A.1 (ATCC^®^ TIB-67). To determine the cytotoxic concentration fifty (CC_50_), the concentration of a compound that inhibits 50% of the cell population, a colorimetric method, based on the New York Cell Viability Reagent (Thermo Fisher Scientific, Waltham, MA, USA), was used.

In 96-well plates, 10^4^ macrophages in 50 µL of RPMI medium were added to each well. Following complete adherence of the cells, serial dilutions of the compounds were added with 10% alamarBlue^TM^. The plates were incubated for 24 h at 37 °C in a 5% CO_2_ atmosphere. After 24 h of incubation, the plates were read using the EnSpire Multimode Plate Reader^®^ at an excitation wavelength of 544 nm and an emission wavelength of 590 nm to determine the CC_50_.

Statistical Analysis

The inhibitory concentration fifty (CC_50_) was determined through the nonlinear regression analysis “[Inhibitor] vs. response—Variable slope (four parameters)”, using GraphPad Prism 10.1.1. Each experiment was conducted in triplicate, and average values were reported. The results were expressed as mean ± standard deviation. Statistical significance was evaluated using a paired two-tailed t-test, with *p* values below 0.05 considered statistically significant.

## 5. Conclusions

Our findings highlight the chemical and biological significance of the four bibenzyl derivatives isolated from *Radula voluta*. Compounds **3** and **4** are reported here for the first time from this species, expanding the known chemical diversity within the genus. The overall antiprotozoal profile of these metabolites reinforces the potential of bibenzyl scaffolds as promising antiparasitic templates. Among the isolates, compounds **2** and **4** showed the highest activity, likely influenced by the presence of isoprenoid side chains and free phenolic hydroxyl groups, which enhance lipophilicity and membrane interaction. Compound **1** exhibited the best selectivity index, suggesting that subtle structural variations within this class can markedly affect biological performance. Together, these results support the notion that *R. voluta* is a valuable natural source of structurally diverse bibenzyls and provide a foundation for future semi-synthetic modification aimed at optimizing potency and selectivity against protozoan pathogens.

## Figures and Tables

**Table 1:** Activity of compounds **1**–**4** isolated from *Radula voluta*; the results demonstrate the activity of each compound at two concentrations, 25 and 50 µg/mL.

Parasite	*T cruzi*		*L. amazonensis*		*L. donovani*		*N. fowleri*		*A. castellanii* Neff	
Concentration (µg/mL)	25	50	25	50	25	50	25	50	25	50
**1**	-	+	+	+	-	-	+	+	-	+
**2**	+	+	+	+	-	+	+	+	-	-
**3**	-	+	-	+	-	-	+	+	-	+
**4**	+	+	+	+	-	+	+	+	-	+

The symbol (+) indicates that the parasites are sensitive to the compound at this concentration; the symbol (-) indicates no activity of compounds at this concentration.

**Table 2:** Cytotoxicity against murine macrophages of compounds **1**–**4** isolated from *Radula voluta*; the results are expressed as CC_50_ ± SD in µg/mL.

Compound	CC_50_
**1**	50.11 ± 12.38
**2**	14.32 ± 0.51
**3**	57.85 ± 10.94
**4**	18.18 ± 2.38

**Table 3:** Results of activity and toxicity of the commercial reference drugs; results were expressed as inhibitory concentration 50 (IC_50_) and cytotoxic concentration 50 (CC_50_) ± standard deviation (SD) [30,31,32].

Drug	Target Organism/Cell Line	Parameter	Mean ± SD (µg/mL)
Miltefosine	*L. donovani*	IC_50_	1.35 ± 0.11
	*L. amazonensis*	IC_50_	2.64 ± 0.10
	*J774A.1 macrophages*	CC_50_	29.43 ± 3.61
Benznidazole	*T. cruzi*	IC_50_	1.80 ± 0.20
	*J774A.1 macrophages*	CC_50_	104.08 ± 0.36
Chlorhexidine	*A. castellanii* Neff	IC_50_	1.53 ± 0.89
	*J774A.1 macrophages*	CC_50_	15.11 ± 0.19
Voriconazole	*A. castellanii* Neff	IC_50_	0.35 ± 0.04
	*J774A.1 macrophages*	CC_50_	7.56 ± 2.20
Amphotericin B	*N. fowleri*	IC_50_	0.16 ± 0.01
	*J774A.1 macrophages*	CC_50_	>200

## Data Availability

Data are available from the authors upon reasonable request.

## Supplementary Materials

The following supporting information can be downloaded at: https://www.mdpi.com/article/10.3390/molecules30234543/s1, Table S1. ^1^H NMR and ^13^C NMR data of compounds (**1–4**) in CDCl_3_; Figure S1. ^1^H NMR data of 2-prenyl-3,5 dihydroxy-bibenzyl (**1**) in CDCl_3_; Figure S2. ^13^C NMR data of 2-prenyl-3,5 dihydroxy-bibenzyl (**1**) in CDCl_3_; Figure S3. HSQC of 2-prenyl-3,5 dihydroxy-bibenzyl (**1**) in CDCl_3_; Figure S4. HMBC of 2-prenyl-3,5 dihydroxy-bibenzyl (**1**) in CDCl_3_; Figure S5. ^1^H NMR data of 2-geranyl-3,5-dihydroxy-bibenzyl (**2**) in CDCl_3_; Figure S6. ^13^C NMR data of 2-geranyl-3,5-dihydroxy-bibenzyl (**2**) in CDCl_3_; Figure S7. HSQC of 2-geranyl-3,5-dihydroxy-bibenzyl (**2**) in CDCl_3_; Figure S8. HMBC of 2-geranyl-3,5-dihydroxy-bibenzyl (**2**) in CDCl_3_; Figure S9. ^1^H NMR data of 2,2-dimethyl-5-phenethyl-2H-chromen-7-ol (**3**) in CDCl_3_; Figure S10. ^13^C NMR data of 2,2-dimethyl-5-phenethyl-2H-chromen-7-ol (**3**) in CDCl_3_; Figure S11. HSQC of 2,2-dimethyl-5-phenethyl-2H-chromen-7-ol (**3**) in CDCl_3_; Figure S12. HMBC of 2,2-dimethyl-5-phenethyl-2H-chromen-7-ol (**3**) in CDCl_3_; Figure S13. ^1^H NMR data of radulanin L (**4**) in CDCl_3_; Figure S14. ^13^C NMR data of radulanin L (**4**) in CDCl_3_ (126 MHz); Figure S15. HSQC of radulanin L (**4**) in CDCl_3_; Figure S16. HMBC of radulanin L (**4**) in CDCl_3_; S21: SwissADME analysis of 2-prenyl-3,5 dihydroxy-bibenzyl (**1**); S22: SwissADME analysis of 2-geranyl-3,5-dihydroxy-bibenzyl (**2**); S23: SwissADME analysis of 2,2-dimethyl-5-phenethyl-2H-chromen-7-ol (**3**); S24: SwissADME analysis of radulanin L (**4**): S25: BOILED-Egg Model of compounds **1**–**4.** Refs. [41,42] are cited in the Supplementary Materials.

## References

[1] Liu Q., Liu M., Liang W., Li X., Jing W., Chen Z., Liu J. (2025). Global distribution and health impact of infectious disease outbreaks, 1996–2023: A worldwide retrospective analysis of World Health Organization emergency event reports. J. Glob. Health.

[2] Sangenito L.S., da Silva Santos V., d’Avila-Levy C.M., Branquinha M.H., Souza Dos Santos A.L., de Oliveira S.S.C. (2019). Leishmaniasis and Chagas Disease—Neglected Tropical Diseases: Treatment Updates. Curr. Top. Med. Chem..

[3] Pana A., Vijayan V., Anilkumar A.C. (2023). Amebic Meningoencephalitis. StatPearls.

[4] Raghavan A., Rammohan R. (2024). Acanthamoeba keratitis—A review. Indian J. Ophthalmol..

[5] Gradstein S.R., William R. (2021). The Liverworts and Hornworts of Colombia and Ecuador.

[6] Gradstein S.R. (2020). Checklist of the Liverworts and hornworts of Ecuador. Frahmia.

[7] Jensen S., Omarsdottir S., Bwalya A.G., Nielsen M.A., Tasdemir D., Olafsdottir E.S. (2012). Marchantin A, a macrocyclic bisbibenzyl ether, isolated from the liverwort Marchantia polymorpha, inhibits protozoal growth in vitro. Phytomedicine.

[8] Pannequin A., Quetin-Leclercq J., Costa J., Tintaru A., Muselli A. (2023). First phytochemical profiling and in-vitro antiprotozoal activity of essential oil and extract of *Plagiochila porelloides*. Molecules.

[9] Ludwiczuk A., Nagashima F., Gradstein R.S., Asakawa Y. (2008). Volatile components from selected Mexican, Ecuadorian, Greek, German and Japanese liverworts. Nat. Prod. Commun..

[10] Asakawa Y., Ludwiczuk A., Nagashima F., Toyota M., Hashimoto T., Tori M., Harinantenaina L. (2009). Bryophytes: Bio-and chemical diversity, bioactivity and chemosystematics. Heterocycles.

[11] Valarezo E., Tandazo O., Galán K., Rosales J., Benítez Á. (2020). Volatile metabolites in Liverworts of Ecuador. Metabolites.

[12] Morocho V., Benitez Á., Carrión B., Cartuche L. (2024). Novel study on chemical characterization and antimicrobial, antioxidant, and anticholinesterase activity of essential oil from Ecuadorian bryophyte *Syzygiella rubricaulis* (Nees) Stephani. Plants.

[13] Toyota M., Kinugawa T., Asakawa Y. (1994). Bibenzyl cannabinoid and bisbibenzyl derivative from the liverwort *Radula perrottetii*. Phytochemistry.

[14] Asakawa Y., Nagashima F., Ludwiczuk A. (2020). Distribution of bibenzyls, prenyl bibenzyls, bis-bibenzyls, and terpenoids in the liverwort genus *Radula*. J. Nat. Prod..

[15] Asakawa Y., Toyota M., Nakaishi E., Tada Y. (1996). Distribution of terpenoids and aromatic compounds in New Zealand liverworts. J. Hattori Bot. Lab..

[16] Zhang C.Y., Gao Y., Zhu R.X., Qiao Y.N., Zhou J.C., Zhang J.Z., Li Y., Li S.W., Fan S.H., Lou H.X. (2019). Prenylated bibenzyls from the Chinese Liverwort *Radula constricta* and their mitochondria-derived paraptotic cytotoxic activities. J. Nat. Prod..

[17] Kinghorn A., Falk O.H., Kobayashi L.J. (2013). Chemical Constituents of Bryophyta. Chemical Constituents of Bryophytes. Bio- and Chemical Diversity, Biological Activity, and Chemosystematics.

[18] Mues R., Zinsmeister H.D. (1988). The chemotaxonomy of phenolic compounds in bryophytes. J. Hattori Bot. Lab..

[19] Lorimer S.D., Perry N.B., Tangney R.S. (1993). An antifungal bibenzyl from the New Zealand liverwort, *Plagiochzla stephensonzana*. Synthesis, and analysis Bioactivity-directed isolation. J. Nat. Prod..

[20] Iwai Y., Murakami K., Gomi Y., Hashimoto T., Asakawa Y., Okuno Y., Ishikawa T., Hatakeyama D., Echigo N., Kusuhara T. (2011). Anti-influenza activity of marchantins, macrocyclic bisbibenzyls contained in liverworts. PLoS ONE.

[21] Nandy S., Dey A. (2020). Bibenzyls and bisbibenzyls of bryophytic origin as promising source of novel therapeutics: Pharmacology, synthesis and structure-activity. J. Pharm. Sci..

[22] Asakawa Y., Hashimoto T., Takikawa K., Tori M., Ogawa S. (1991). Prenyl bibenzyls from the liverworts *Radula perrottetii* and *Radula complanata*. Phytochemistry.

[23] Labbé C., Faini F., Villagrán C., Coll J., Rycroft D.S. (2007). Bioactive polychlorinated bibenzyls from the liverwort *Riccardia polyclada*. J. Nat. Prod..

[24] Otoguro K., Ishiyama A., Iwatsuki M., Namatame M., Tukashima A.N., Kiyohara H., Hashimoto T., Asakawa Y., Omura S., Yamada H. (2012). In vitro antitrypanosomal activity of bis(bibenzyls) and bibenzyls from liverworts against *Trypanosoma brucei*. J. Nat. Med..

[25] Nagashima F., Momosaki S., Watanabe Y., Toyota M., Huneck S., Asakawat Y. (1996). Terpenoids and aromatic compounds from six liverworts. Phytochemistry.

[26] Kraut L., Must R., Dietmar Z.H. (1997). Prenylated bibenzyl derivatives from *Lethocolea glossophylla* and *Radula voluta*. Phytochemistry.

[27] Nagashima F., Asakawa Y. (2011). Terpenoids and bibenzyls from three Argentine liverworts. Molecules.

[28] Asakawa Y., Kondo K., Tori M. (1991). Cyclopropanochroman derivatives from the liverwort *Radula javanica*. Phytochemistry.

[29] Asakawa Y., Kondo K., Takikawa E.K., Tori M., Hashimoto T., Ogawa S. (1991). Prenyl bibenzyls from the liverworts *Radula kojana*. Phytochemistry.

[30] Bethencourt-Estrella C.J., López-Arencibia A., Lorenzo-Morales J., Piñero J.E. (2024). Global Health Priority Box: Discovering Flucofuron as a Promising Antikinetoplastid Compound. Pharmaceuticals.

[31] Rodríguez-Expósito R.L., Nicolás-Hernández D.S., Sifaoui I., Cuadrado C., Salazar-Villatoro L., Reyes-Batlle M., Hernández-Daranas A., Omaña-Molina M., Fernández J.J., Díaz-Marrero A.R. (2023). Gongolarones as antiamoeboid chemical scaffold. Biomed. Pharmacother..

[32] Chao-Pellicer J., Arberas-Jiménez I., Delgado-Hernández S., Sifaoui I., Tejedor D., García-Tellado F., Piñero J.E., Lorenzo-Morales J. (2023). Cyanomethyl Vinyl Ethers Against *Naegleria fowleri*. ACS Chem. Neurosci..

[33] Stuart K., Brun R., Croft S., Fairlamb A., Gütteridge W., McKerrow J., Reed S., Tarleton R. (2008). Kinetoplastids: Related protozoan pathogens, different diseases. J. Clin. Investig..

[34] Scorza B.M., Carvalho E.M., Wilson M.E. (2017). Cutaneous manifestations of human and murine leishmaniasis. Int. J. Mol. Sci..

[35] Grace E., Asbill S., Virga K. (2015). Naegleria fowleri: Pathogenesis, diagnosis, and treatment options. Antimicrob. Agents Chemother..

[36] Asakawa Y. (2007). Biologically active compounds from bryophytes. Pure Appl. Chem..

[37] Rosa L.H., Furtado G.P., Barata L.E.S. (2011). Antimicrobial activity of compounds isolated from liverworts: A review. Phytomedicine.

[38] Roldos V., Nakayama H., Rolón M., Montero-Torres A., Truccu F., Torres S., Vega C., Marrero-Ponce Y., Heguaburu V., Yaluff G. (2008). Activity of a hydroxybibenzyl bryophyte constituent against *Leishamania* spp. and *Trypanosoma cruzi:* In silico, in vitro and in vivo activity studies. Eur. J. Med. Chem..

[39] Cos P., Maes L., Vlietinck A.J., Berghe D.V. (2004). Plant-derived leading compounds for chemotherapy of human protozoan infections. Planta Med..

[40] Siddiqui R., Khan N.A. (2014). Biology and pathogenesis of *Naegleria fowleri*. Acta Trop..

[41] Daina A., Michielin O., Zoete V. (2017). SwissADME: A free web tool to evaluate pharmacokineZcs, drug-likeness and medicinal chemistry friendliness of small molecules. Sci. Rep..

[42] Daina A., Zoete V. (2016). A BOILED-Egg To Predict GastrointesZnal AbsorpZon and Brain PenetraZon of Small Molecules. ChemMedChem.

[43] Asakawa Y., Ludwiczuk A., Nagashima F. (2013). Biologically Active Compounds of the Marchantiophyta and Bryophyta. Chemical Constituents of Bryophytes: Bio- and Chemical Diversity, Biological Activity, and Chemosystematics.

[44] Zhang X., Liu J., Liang J., Wang Z., Wang C. (2021). Structure-activity relationships of prenylated natural products with antiprotozoal activity. Molecules.

[45] Cartuche L., Sifaoui I., López-Arencibia A., Bethencourt-Estrella C.J., San Nicolás-Hernández D., Lorenzo-Morales J., Piñero J.E., Díaz-Marrero A.R., Fernández J.J. (2020). Antikinetoplastid Activity of Indolocarbazoles from *Streptomyces sanyensis*. Biomolecules.

[46] Cartuche L., Reyes-Batlle M., Sifaoui I., Arberas-Jiménez I., Piñero J.E., Fernández J.J., Lorenzo-Morales J., Díaz-Marrero A.R. (2019). Antiamoebic Activities of Indolocarbazole Metabolites Isolated from *Streptomyces sanyensis* Cultures. Mar. Drugs.

[47] Rizo-Liendo A., Sifaoui I., Reyes-Batlle M., Chiboub O., Rodríguez-Expósito R.L., Bethencourt-Estrella C.J., San Nicolás-Hernández D., Hendiger E.B., López-Arencibia A., Rocha-Cabrera P. (2019). In Vitro Activity of Statins against *Naegleria fowleri*. Pathogens.

