# Supplementary material for: Bibenzyl Derivatives from Radula voluta (An Ecuadorian Liverwort): Bioprospecting for Antiprotozoal Properties
Source: Molecules. 2025 Nov 25;30(23):4543. doi: 10.3390/molecules30234543 (PMC12693467; doi:10.3390/molecules30234543)
Supplement: Supplementary file 1 [file molecules-30-04543-s001.zip › molecules-3829859-supplementary.pdf]

Supplementary Material

Bibenzyl Derivatives from *Radula voluta* (an Ecuadorian Liverwort): Bioprospecting for Antiprotozoal Properties

José Miguel Andrade[1,2], Carlos J. Bethencourt Estrella[3,4,5], Javier Chao Pellicer[3,4,5], Luis Cartuche[1], Vladimir Morocho[1], Ángel Benítez[6], Rubén L. Rodriguez Expósito[3,4,5], José E. Piñero[3,4,5], Jacob Lorenzo-Morales[3,4,5,*], Ana R. Díaz-Marrero[7,8,*], José J. Fernandez[2,8]

[1] Departamento de Química, Universidad Técnica Particular de Loja (UTPL), Calle Paris s/n y Praga, 110107, Ecuador; jmandrade@utpl.edu.ec (J.M.A.); lecartuche@utpl.edu.ec (L.C.); svmorocho@utpl.edu.ec (V.M.)

[2] Instituto Universitario de Bio-Orgánica Antonio González (IUBO AG), Universidad de La Laguna (ULL), La Laguna, 38206, Spain; alu0101535456@ull.edu.es (J.M.A.); jjfercas@ull.edu.es (J.J.F)

[3] Instituto Universitario de Enfermedades Tropicales y Salud Pública de Canarias, Universidad de La Laguna, Avda. Astrofísico Fco. Sánchez, S/N, 38203 La Laguna, Tenerife, Islas Canarias, Spain; cbethene@ull.edu.es (C.J.B.E); rrodrige@ull.edu.es (R.L.R.E); jchaopel (J.C.P); jpinero@ull.edu.es (J.E.P); jmlorenz@ull.edu.es (J.L.M)

[4] Departamento de Obstetricia y Ginecología, Pediatría, Medicina Preventiva y Salud Pública, Toxicología Medicina Legal y Forense y Parasitología, Universidad de La Laguna, Tenerife, Islas Canarias, Spain

[5] Centro de investigación Biomédica en Red de Enfermedades Infecciosas (CIBERINFEC), Instituto de Salud Carlos III, 28220 Madrid, Spain

[6] Biodiversidad de Ecosistemas Tropicales-BIETROP, Herbario HUTPL, Departamento de Ciencias Biológicas y Agropecuarias, Universidad Técnica Particular de Loja, San Cayetano s/n, 1101608 Loja, Ecuador; arbenitez@utpl.edu.ec (Á. B.)

[7] Instituto de Productos Naturales y Agrobiología (IPNA), Consejo Superior de Investigaciones Científicas (CSIC), Avenida Astrofísico Francisco Sánchez 3, 38206 La Laguna, Tenerife; adiazmar@ull.edu.es (A.R.D.)

[8] Biotecnología Marina, IUBO-ULL, Unidad Asociada al IPNA-CSIC, 38206 La Laguna, Tenerife, Spain

* Correspondence: jmlorenz@ull.edu.es; adiazmar@ull.edu.es

Table of Contents

1	**Table S1.** ^{1}H NMR and ^{13}C NMR data of compounds (**1–4**) in $CDCl_3$	S3-S4
2	**Figure S1.** ^{1}H NMR data of 2-prenyl-3,5 dihydroxy-bibenzyl (**1**) in $CDCl_3$	S5
3	**Figure S2.** ^{13}C NMR data of 2-prenyl-3,5 dihydroxy-bibenzyl (**1**) in $CDCl_3$	S6
4	**Figure S3.** HSQC of 2-prenyl-3,5 dihydroxy-bibenzyl (**1**) in $CDCl_3$	S7
5	**Figure S4.** HMBC of 2-prenyl-3,5 dihydroxy-bibenzyl (**1**) in $CDCl_3$	S8
6	**Figure S5.** ^{1}H NMR data of 2-geranyl-3,5-dihydroxy-bibenzyl (**2**) in $CDCl_3$	S9
7	**Figure S6.** ^{13}C NMR data of 2-geranyl-3,5-dihydroxy-bibenzyl (**2**) in $CDCl_3$	S10
8	**Figure S7.** HSQC of 2-geranyl-3,5-dihydroxy-bibenzyl (**2**) in $CDCl_3$	S11
9	**Figure S8.** HMBC of 2-geranyl-3,5-dihydroxy-bibenzyl (**2**) in $CDCl_3$	S12
10	**Figure S9.** ^{1}H NMR data of 2,2-dimethyl-5-phenethyl-2H-chromen-7-ol (**3**) in $CDCl_3$	S13
11	**Figure S10.** ^{13}C NMR data of 2,2-dimethyl-5-phenethyl-2H-chromen-7-ol (**3**) in $CDCl_3$	S14
12	**Figure S11.** HSQC of 2,2-dimethyl-5-phenethyl-2H-chromen-7-ol (**3**) in $CDCl_3$	S15
13	**Figure S12.** HMBC of 2,2-dimethyl-5-phenethyl-2H-chromen-7-ol (**3**) in $CDCl_3$	S16
14	**Figure S13.** ^{1}H NMR data of radulanin L (**4**) in $CDCl_3$	S17
15	**Figure S14.** ^{13}C NMR data of radulanin L (**4**) in $CDCl_3$ (126 MHz)	S18
16	**Figure S15.** HSQC of radulanin L (**4**) in $CDCl_3$	S19
17	**Figure S16.** HMBC of radulanin L (**4**) in $CDCl_3$	S20
18	SwissADME analysis of 2-prenyl-3,5 dihydroxy-bibenzyl (**1**)	S21
19	SwissADME analysis of 2-geranyl-3,5-dihydroxy-bibenzyl (**2**)	S22
20	SwissADME analysis of 2,2-dimethyl-5-phenethyl-2H-chromen-7-ol (**3**)	S23
21	SwissADME analysis of radulanin L (**4**)	S24
22	BOILED-Egg Model of compounds **1-4**	S25

Table S1. ^{1}H NMR and ^{13}C NMR data of compounds (**1–4**) in $CDCl_3$.

	**1**		**2**		**3**		**4**	
**Position**	δ^{1} **H, mult. (*J* in Hz)**	δ^{13} **C**	δ^{1} **H, mult. (*J* in Hz)**	δ^{13} **C**	δ^{1} **H, mult. (*J* in Hz)**	δ^{13} **C**	δ^{1} **H, mult (*J* in Hz)**	δ^{13} **C**
1		142.10		142.22				
2		118.02		117.70		75.77	4.40, br s	74.41
3		156.03		155.88	5.50, d (9.9)	128.10		134.13
4	6.25, d (2.6)	101.80	6.25, d (2.8)	101.54	6.44, d (10.0)	118.87	5.61, t (5.5)	121.03
5		154.81		154.66		139.24	3.40, d (4.0)	21.84
6	6.28, d (2.6)	109.27	6.27, d (2.2)	108.98	6.19, d (2.5)	108.81		152.26
7						156.09	6.39, d (1.6)	111.70
8					6.19, d (2.5)	102.15		141.76
9						154.78	6.55, d (1.6)	113.91
10						113.06		159.85
11								121.03
1´	3.30, d (6.8)	25.26	3.29, d (6.4)	25.03	1.40, s	27.84	1.54, s	20.84
2´	5.10, t (6.0)	123.05	5.11, t (6.0)	122.81	1.40, s	27.84		
3´		134.43		138.10				
4´	1.73, s	26.11	2.03, m	39.78				
5´	1.80, s	18.32	2.09, m	26.57				
6´			5.04, t (6.6)	123.95				
7´				134.20				
8´			1.58, s	16.39				
9´			1.72, s	25.87				
10´			1.79, s	18.08				
α	2.84, s	36.00	2.84, br s	35.78	2.84, br s	34.53	2.83 m	35.84
β	2.84, s	37.92	2.84, br s	37.70	2.84, br s	37.54	2.83 m	32.15
1^{2}		142.51		141.86		141.71		127.50
2^{2}	7.19, m	128.78	7.18, m	128.54	7.18 m	128.54		153.64
3^{2}	7.29, m	128.78	7.29, t (7.5)	128.54	7.28, m	128.54	6.75, dd (8.4, 1.2)	115.54

4²	7.19, m	126.38	7.18, m	126.14	7.18 m	126.16	7.08, m	127.50
5²	7.29, m	128.78	7.29, t (7.5)	128.54	7.28, m	128.54	6.86, ddd (7.5, 7.4, 1.2)	120.85
6²	7.19, m	128.78	7.18, m	128.54	7.18 m	128.54	7.08, m	130.46
OH	5.39, br s						4.87 br s	
OH	5.18, br s		5.34, br s				4.80 br s	

^{1}H- NMR data for compounds 1 - 4, (500 MHz), and 2 - 3 (600 MHz)
^{13}C- NMR data for compounds 1 - 4, (125 MHz), and 2 - 3 (150 MHz)

Figure S1. ^{1}H NMR data of 2-prenyl-3,5 dihydroxy-bibenzyl (**1**) in $CDCl_3$

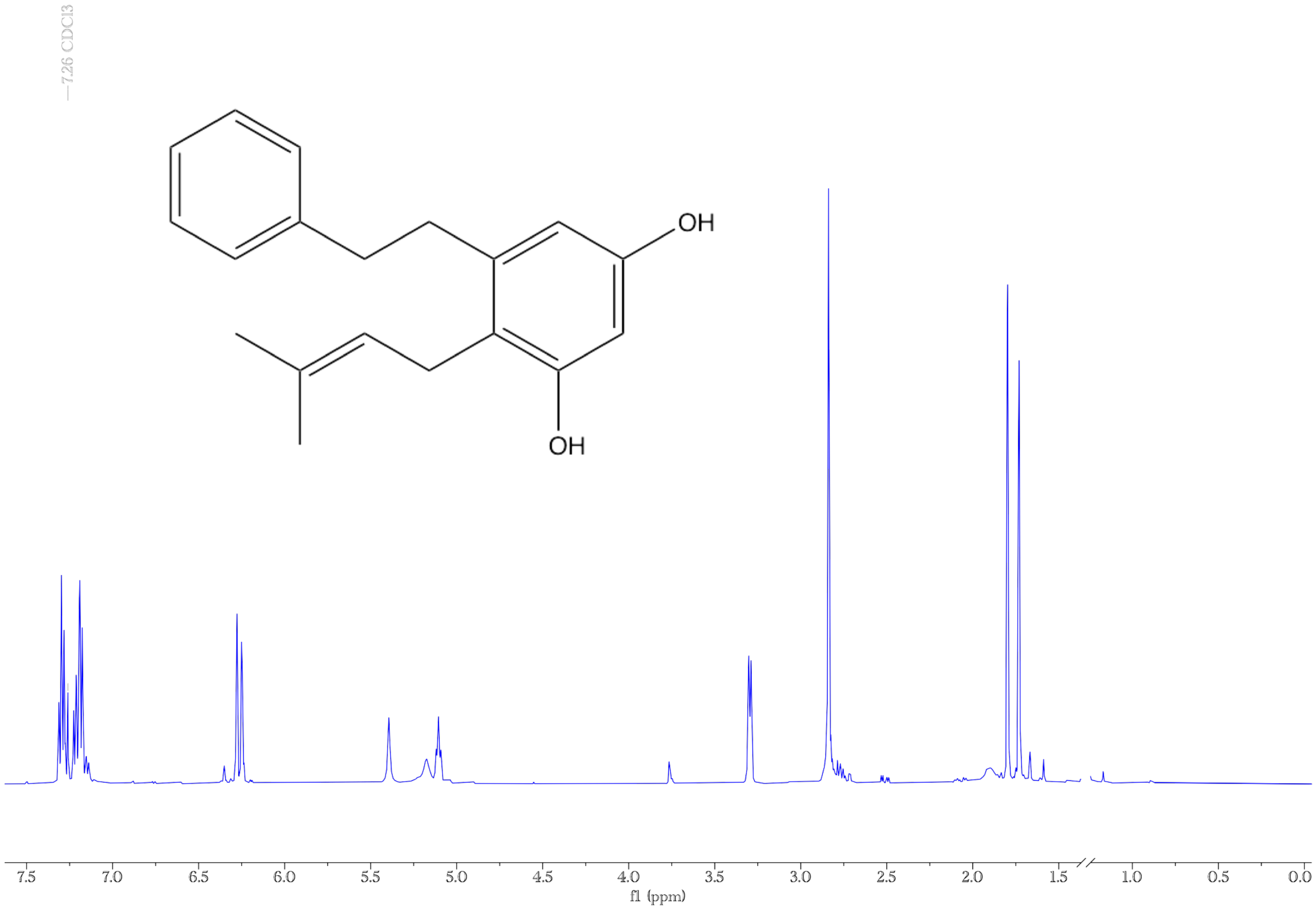

Figure S2. ^{13}C NMR data of 2-prenyl-3,5 dihydroxy-bibenzyl (**1**) in $CDCl_3$

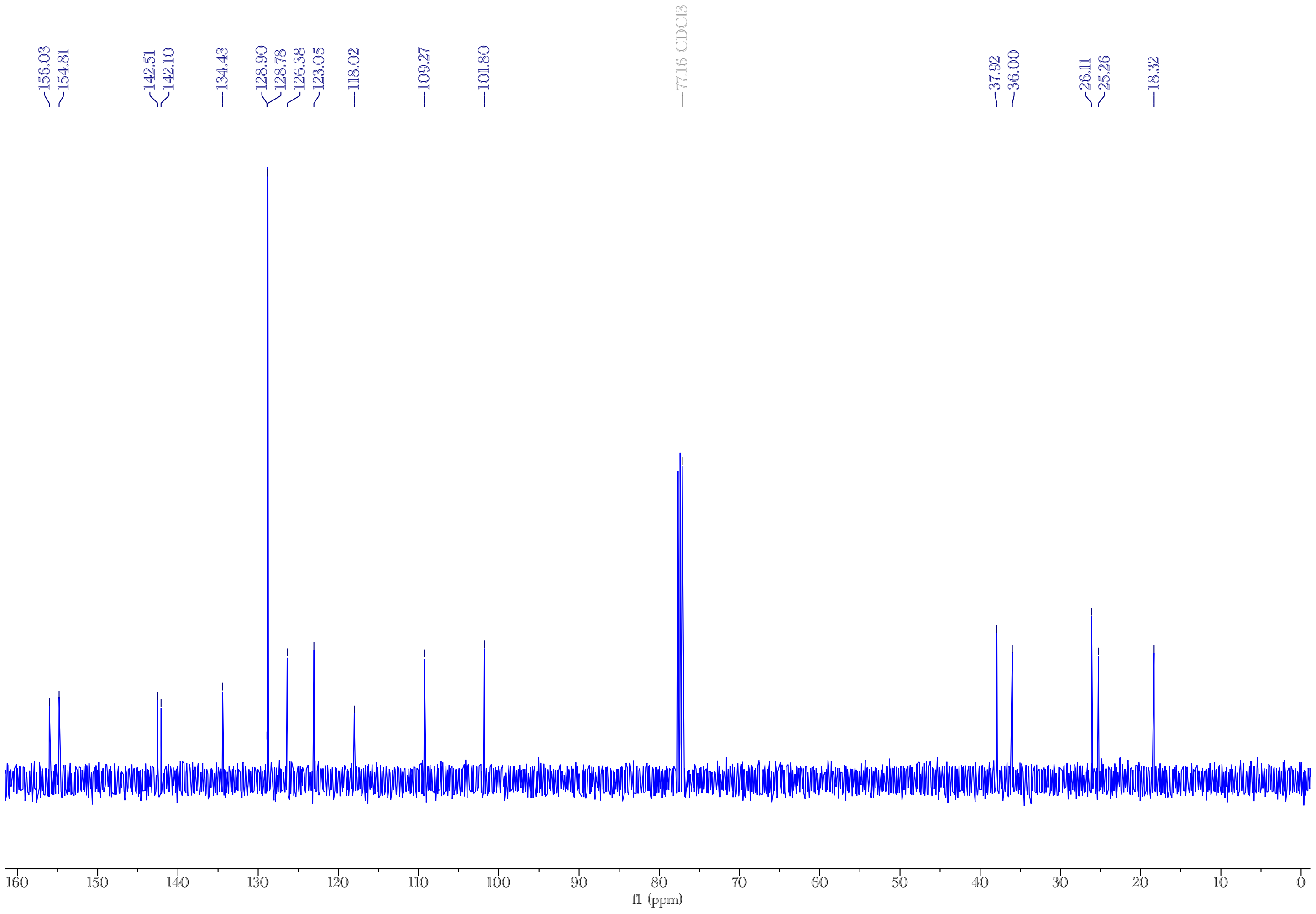

Figure S3. HSQC of 2-prenyl-3,5 dihydroxy-bibenzyl (**1**) in $CDCl_3$

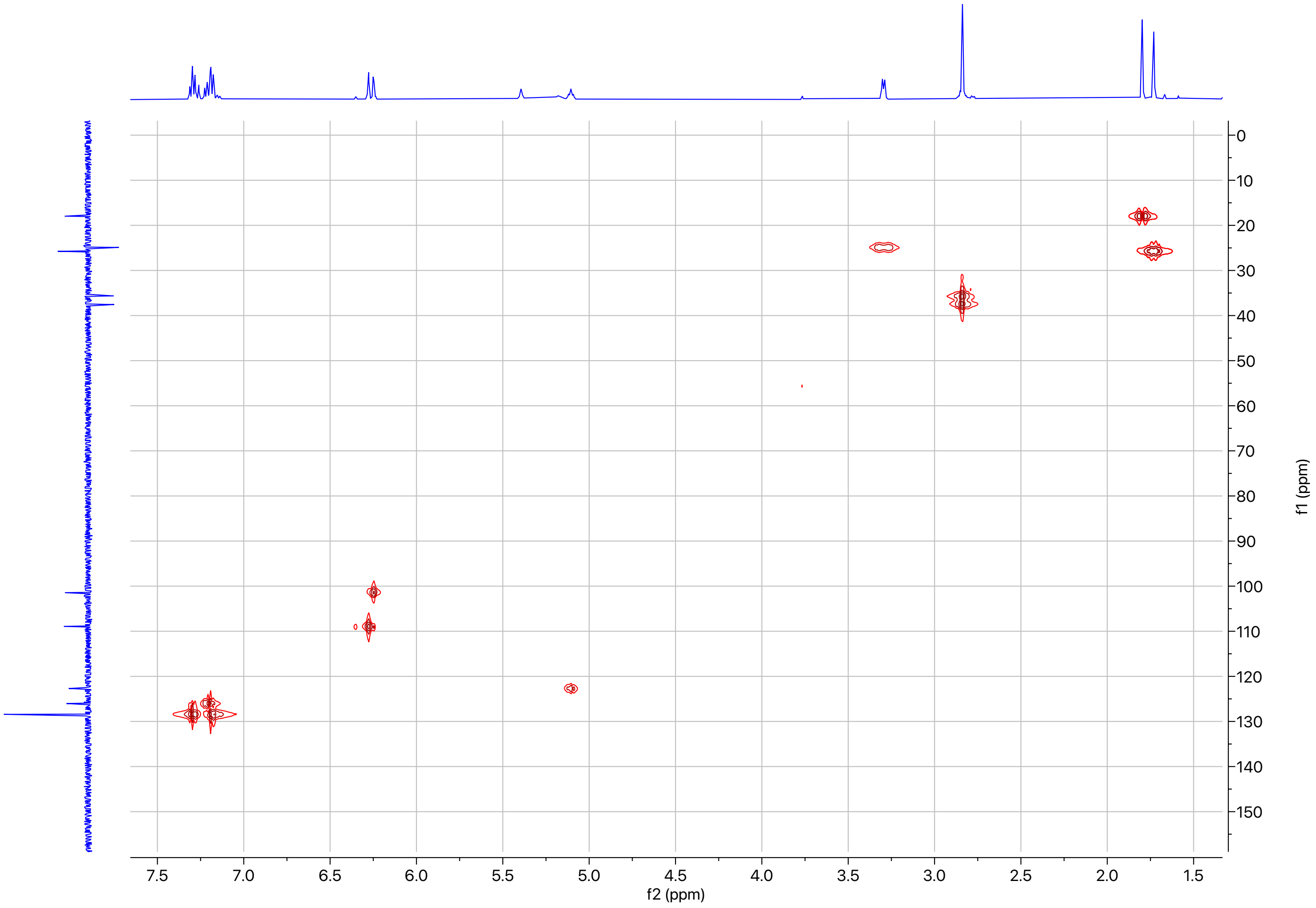

Figure S4. HMBC of 2-prenyl-3,5 dihydroxy-bibenzyl (**1**) in $CDCl_3$

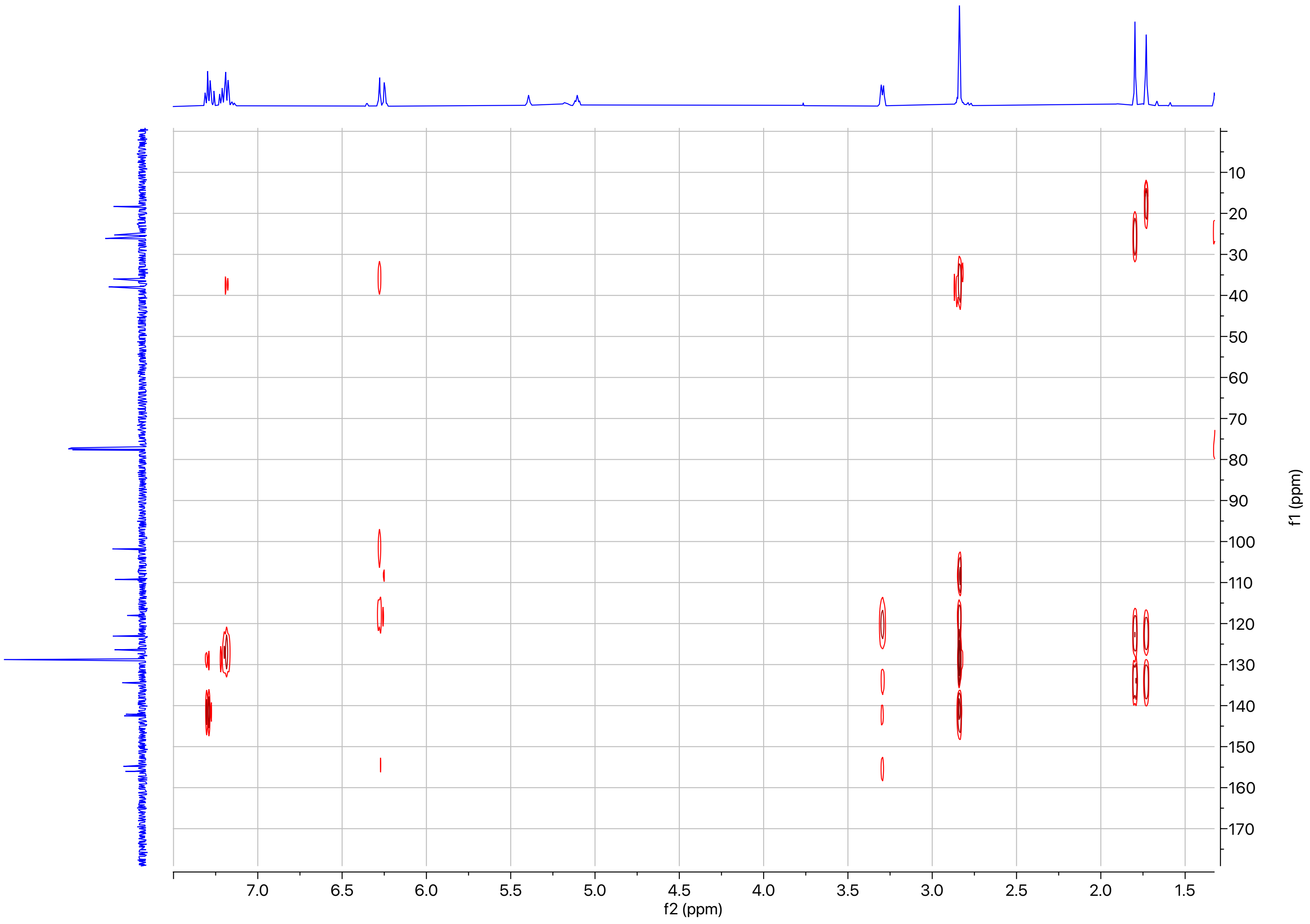

Figure S5. ^{1}H NMR data of 2-geranyl-3,5-dihydroxy-bibenzyl (**2**) in $CDCl_3$

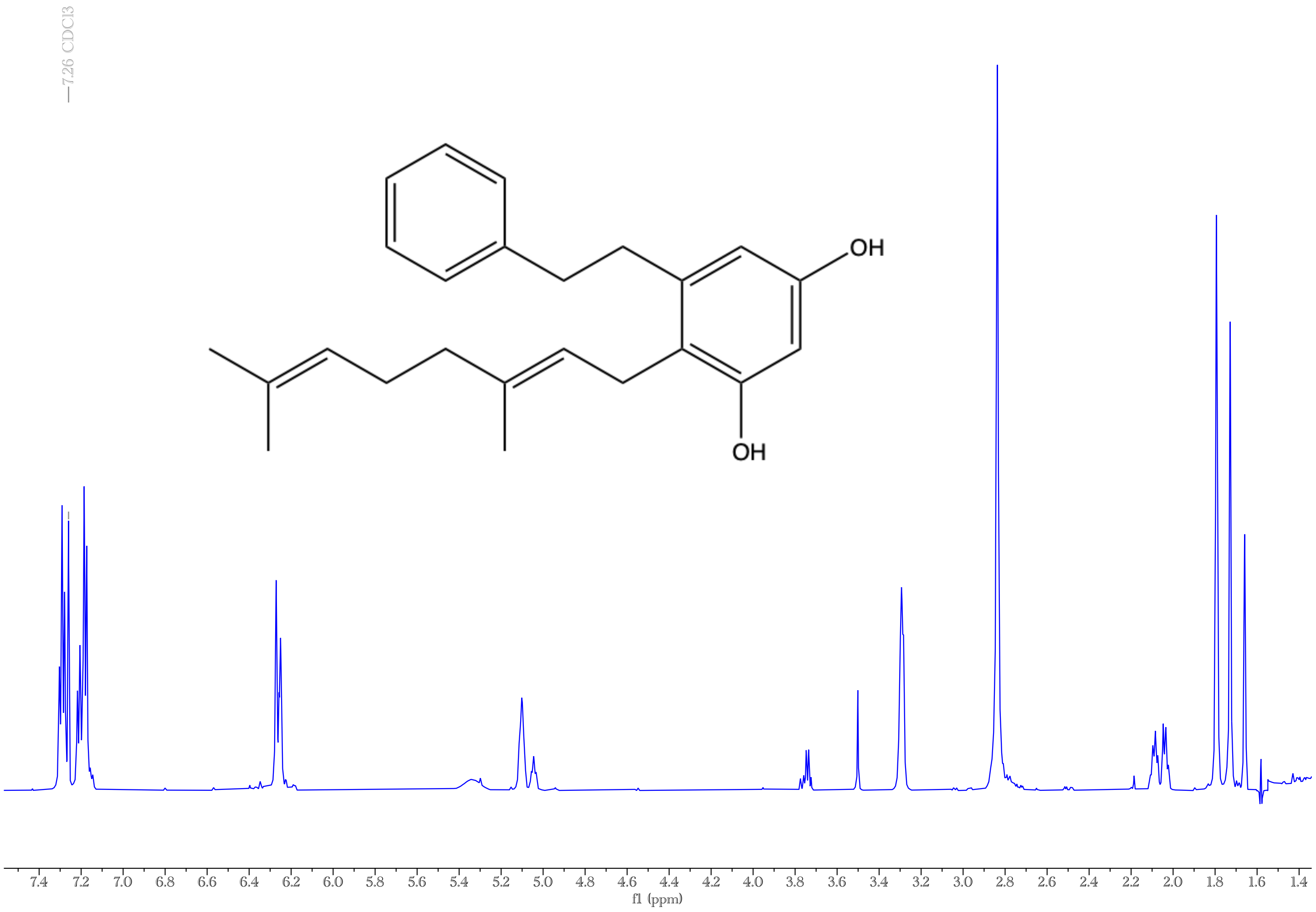

Figure S6. ^{13}C NMR data of 2-geranyl-3,5-dihydroxy-bibenzyl (**2**) in $CDCl_3$

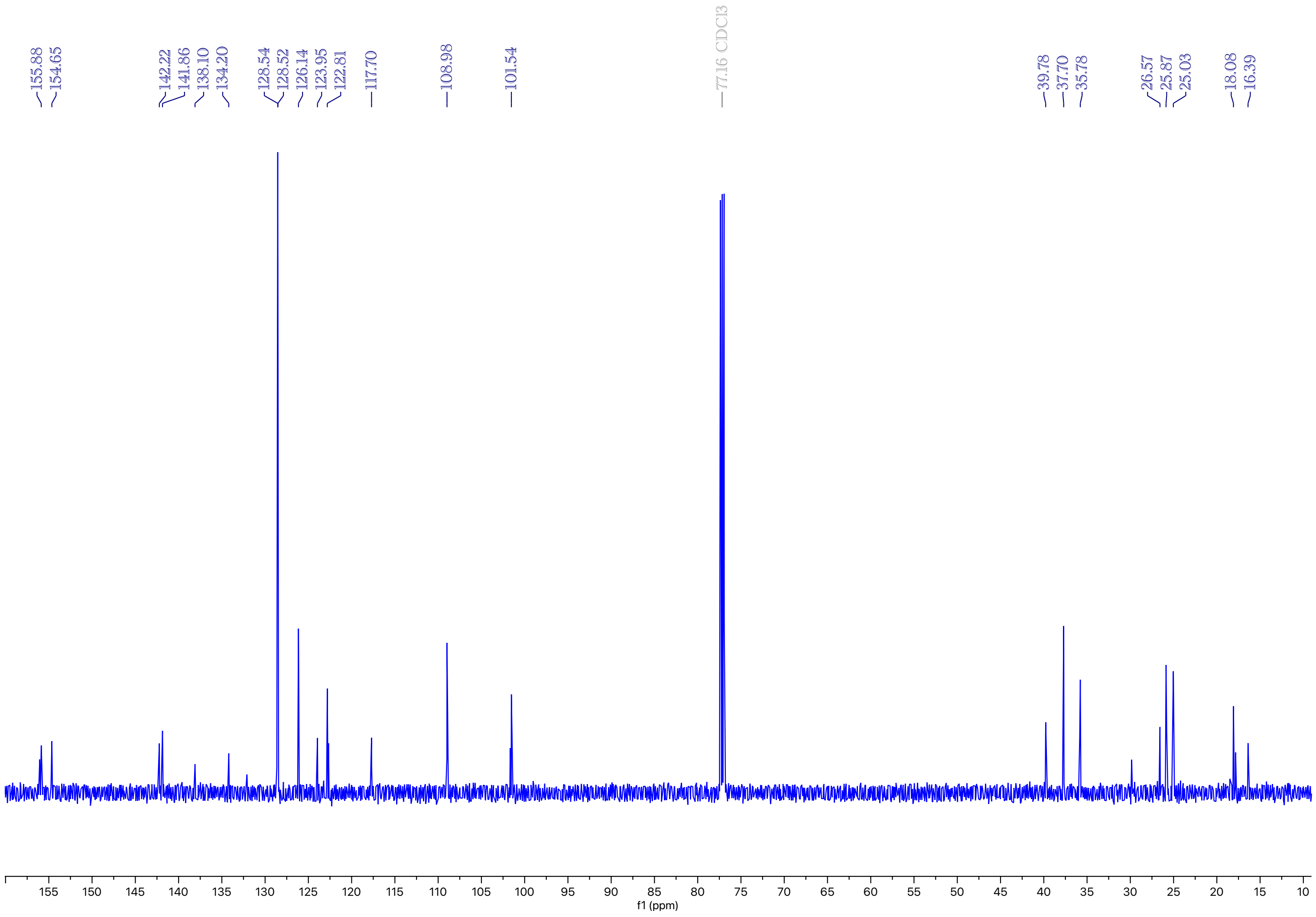

Figure S7. HSQC of 2-geranyl-3,5-dihydroxy-bibenzyl (**2**) in $CDCl_3$

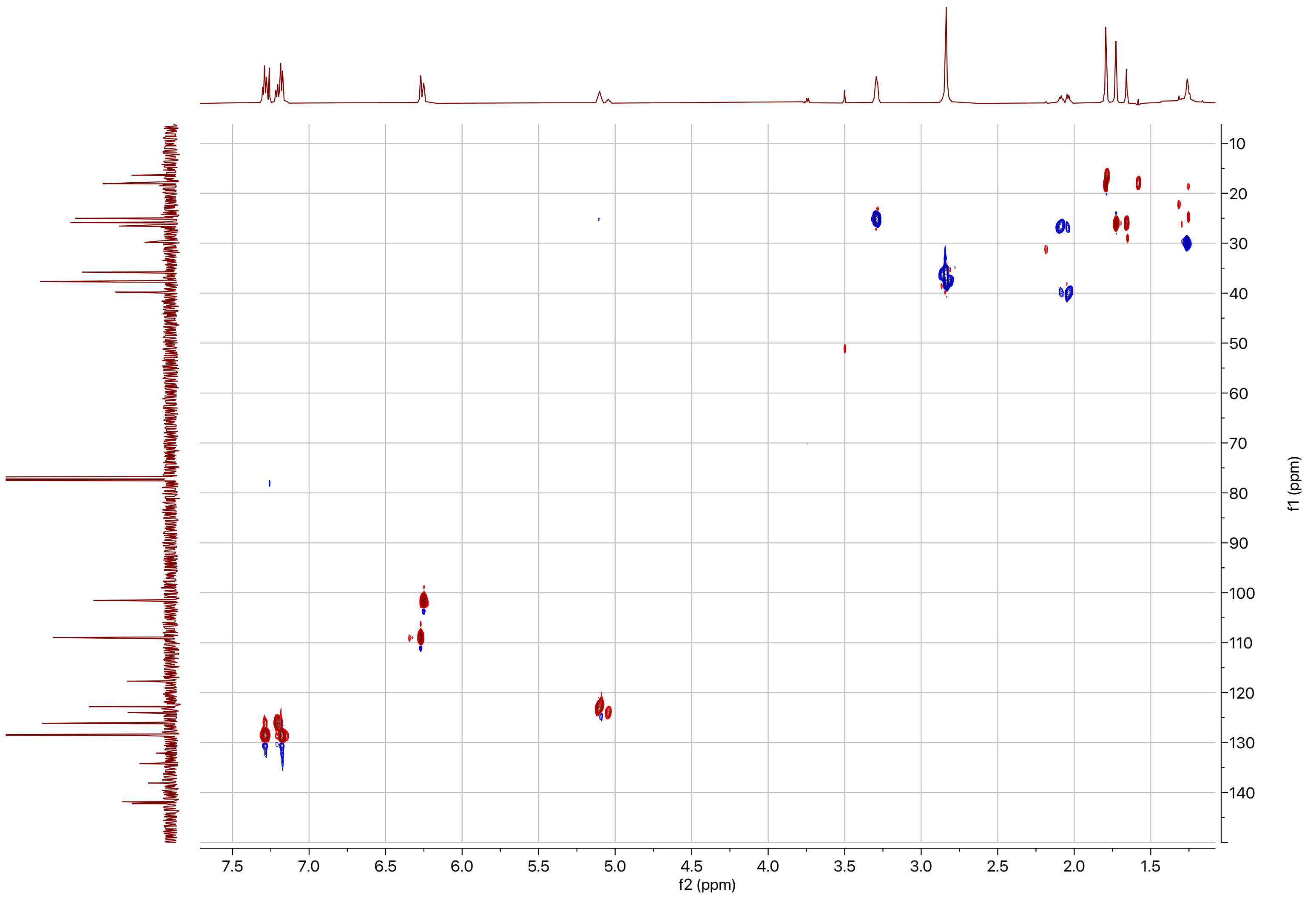

Figure S8. HMBC of 2-geranyl-3,5-dihydroxy-bibenzyl (**2**) in $CDCl_3$

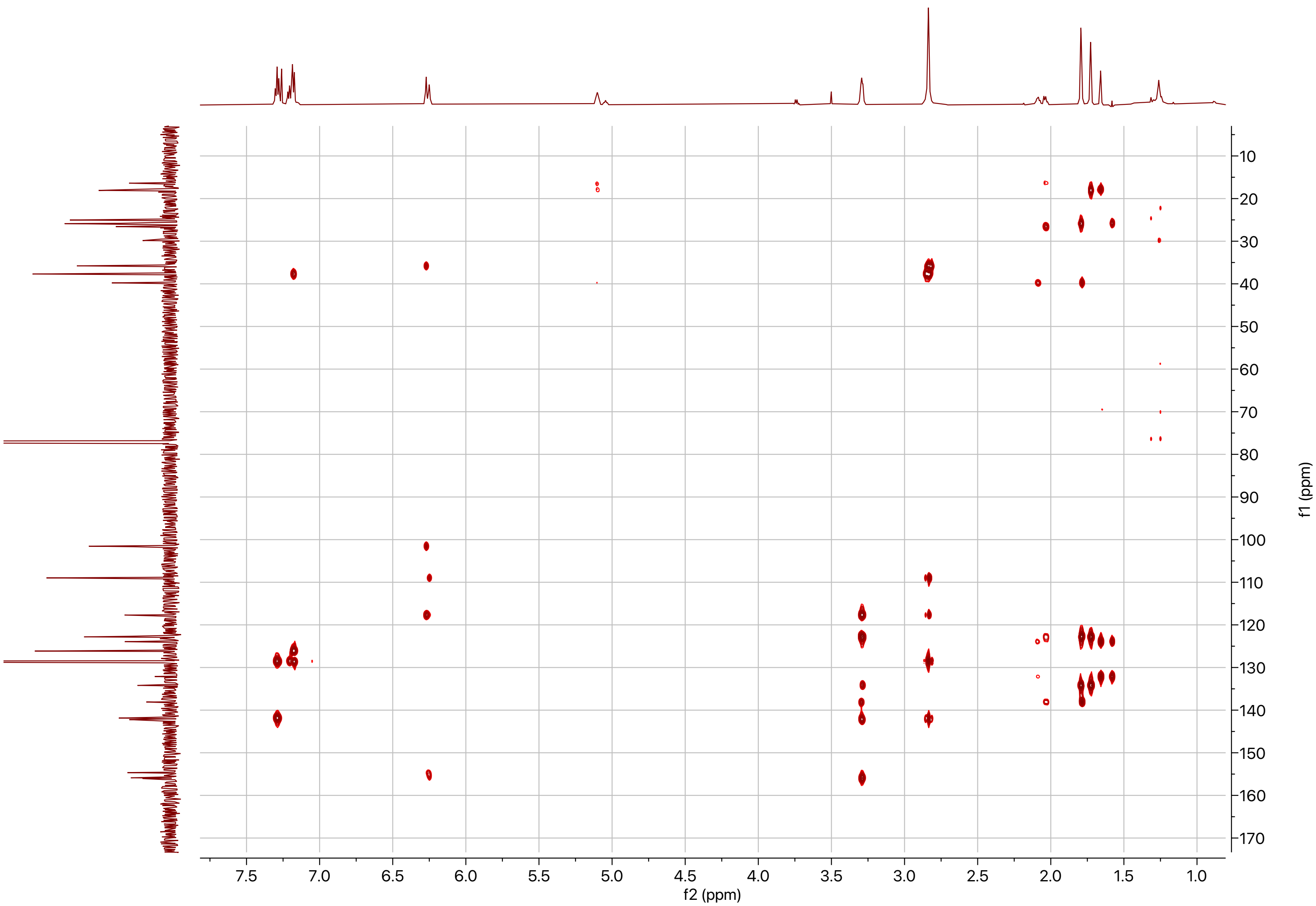

Figure S9. ^{1}H NMR data of 2,2-dimethyl-5-phenethyl-2H-chromen-7-ol (**3**) in $CDCl_3$

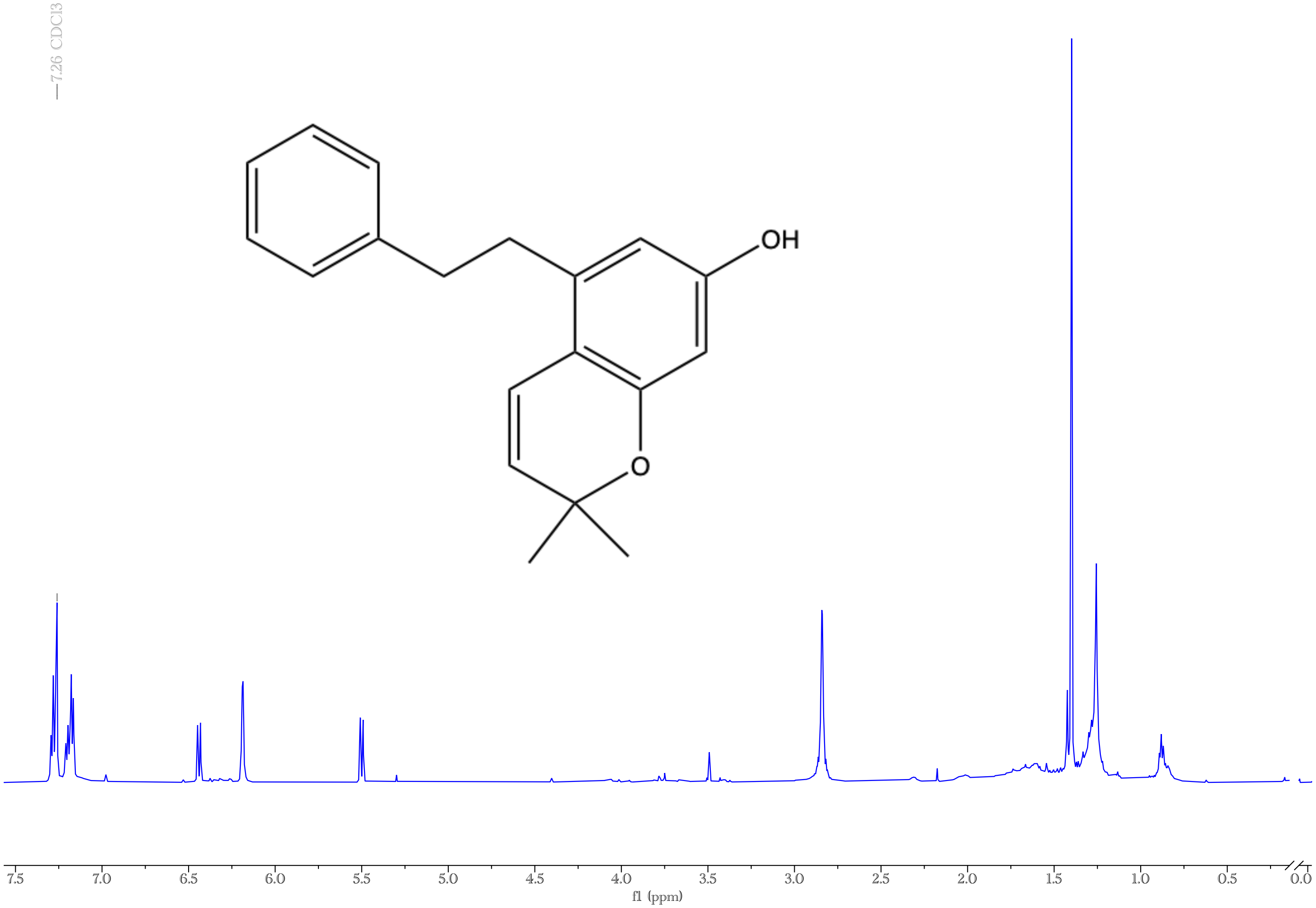

Figure S10. ^{13}C NMR data of 2,2-dimethyl-5-phenethyl-2H-chromen-7-ol (**3**) in $CDCl_3$

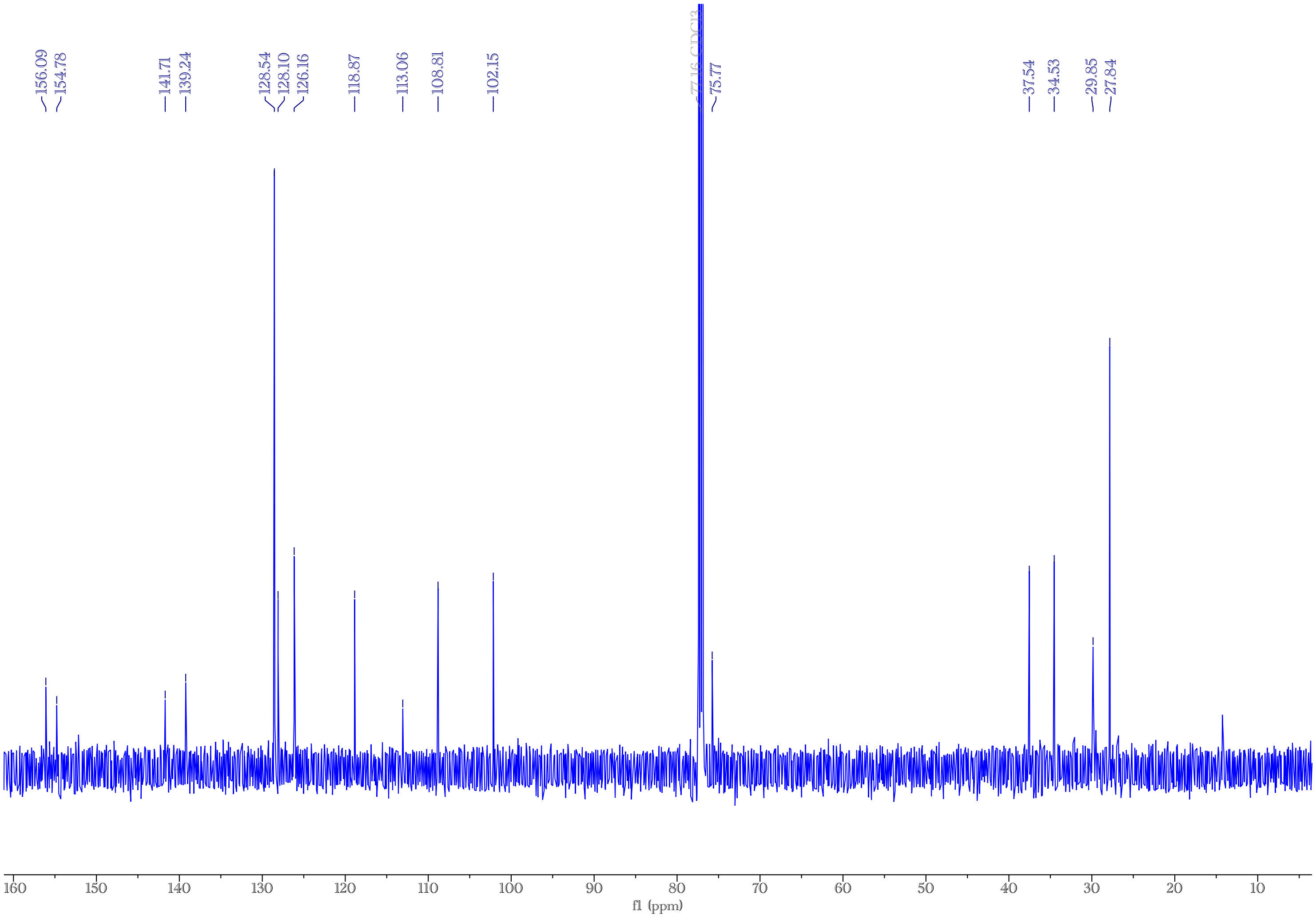

Figure S11. HSQC of 2,2-dimethyl-5-phenethyl-2H-chromen-7-ol (**3**) in $CDCl_3$

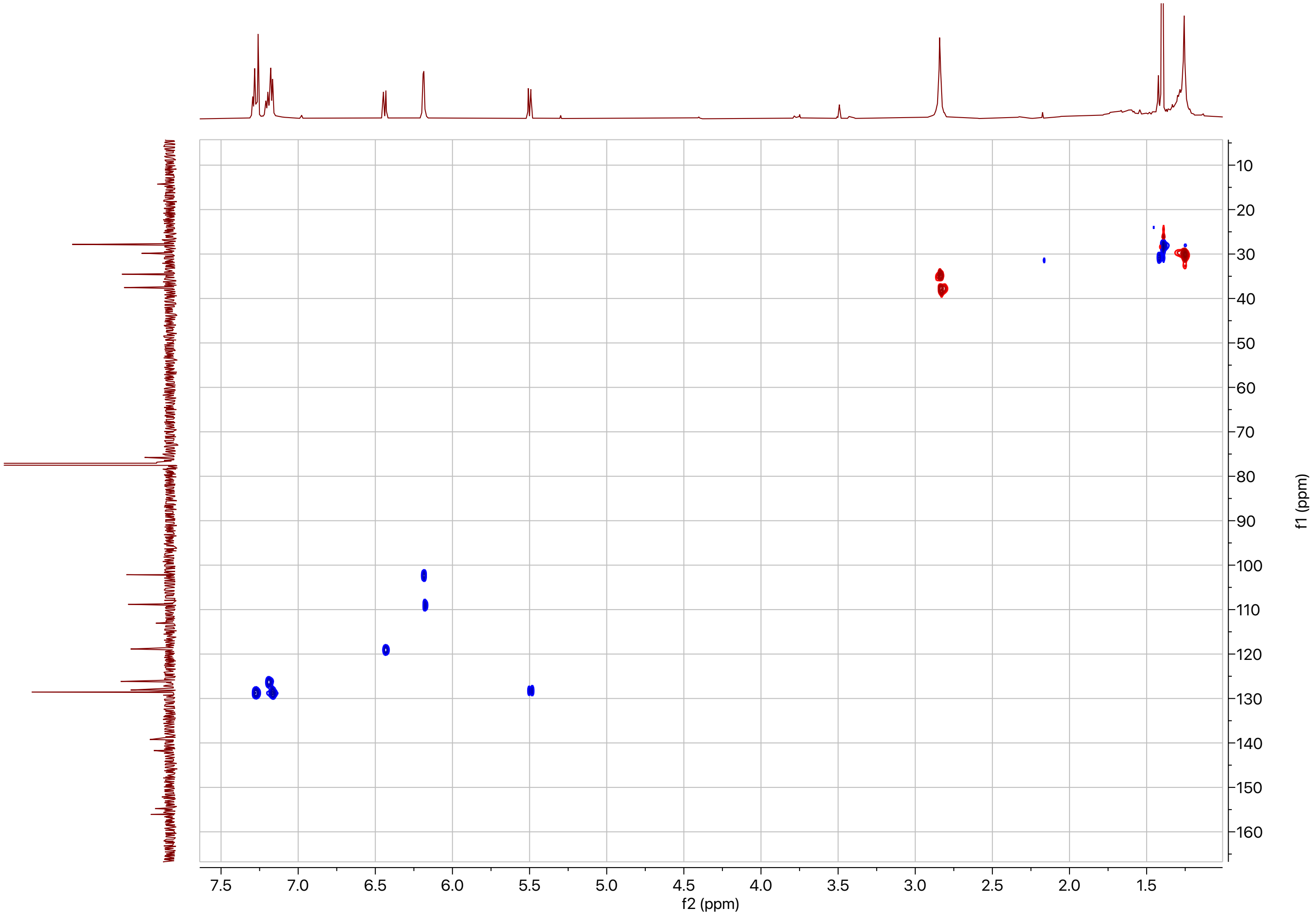

Figure S12. HMBC of 2,2-dimethyl-5-phenethyl-2H-chromen-7-ol (**3**) in $CDCl_3$

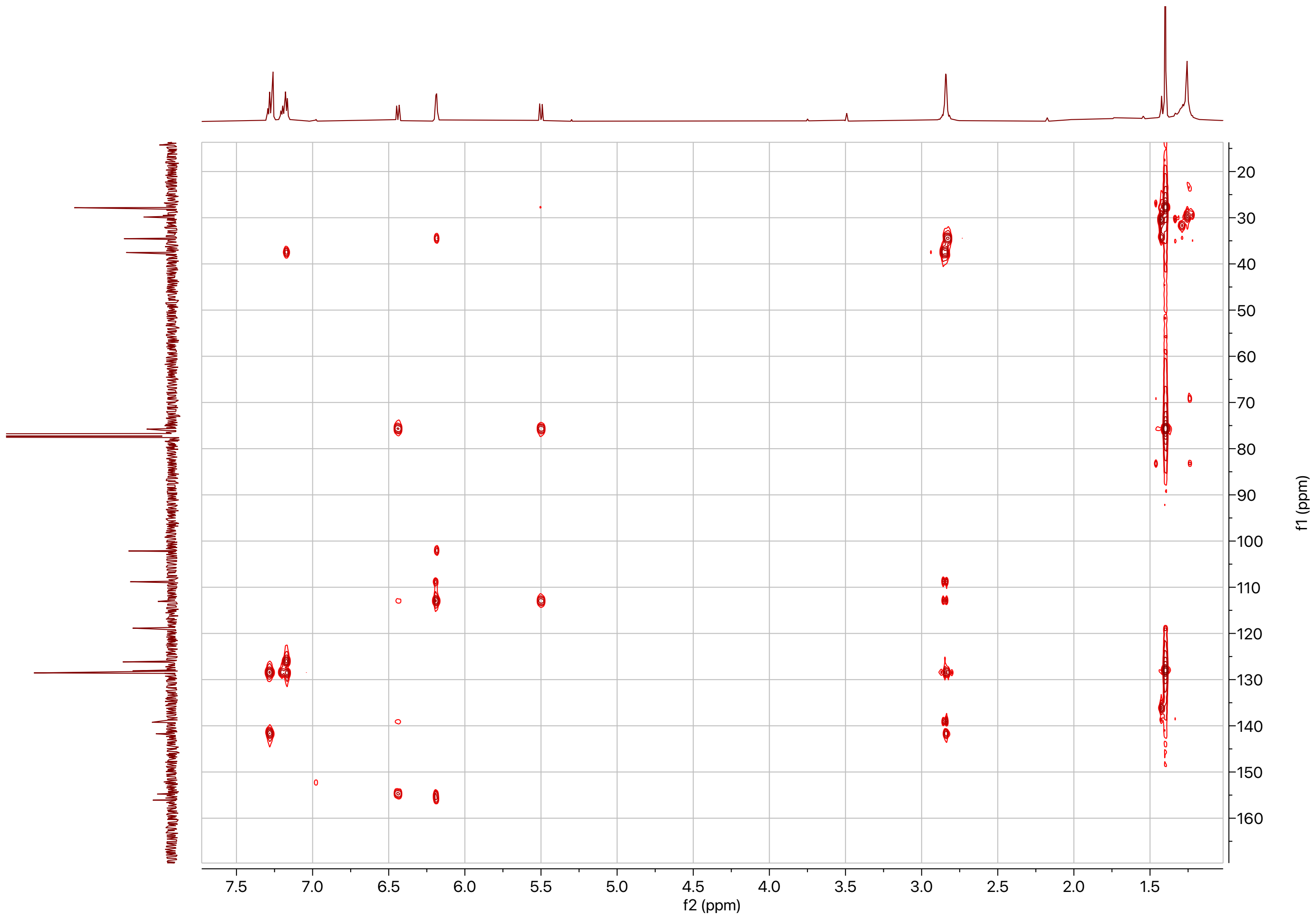

Figure S13. ^{1}H NMR data of radulanin L (**4**) in $CDCl_3$

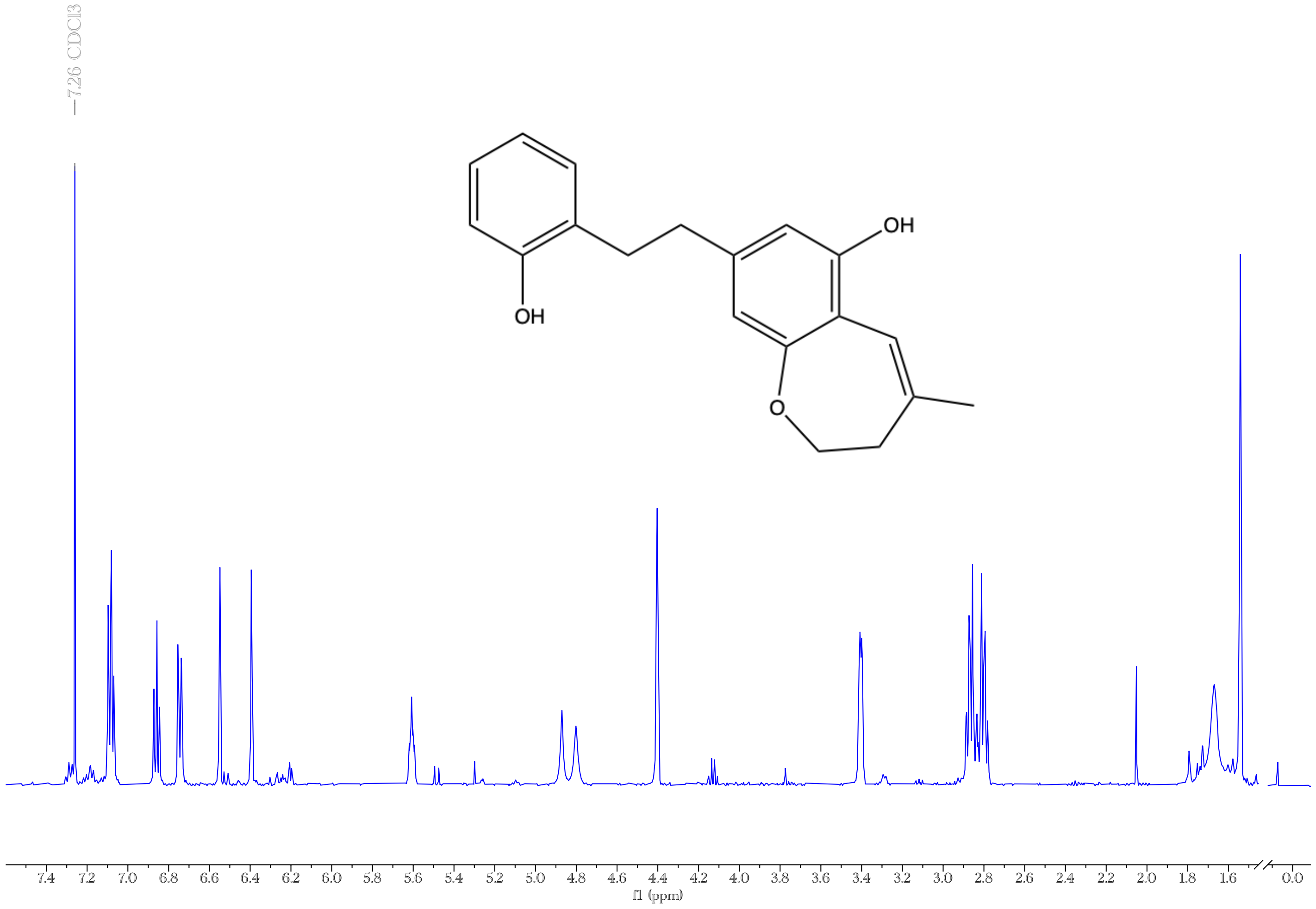

Figure S14. ^{13}C NMR data of radulanin L (**4**) in $CDCl_3$

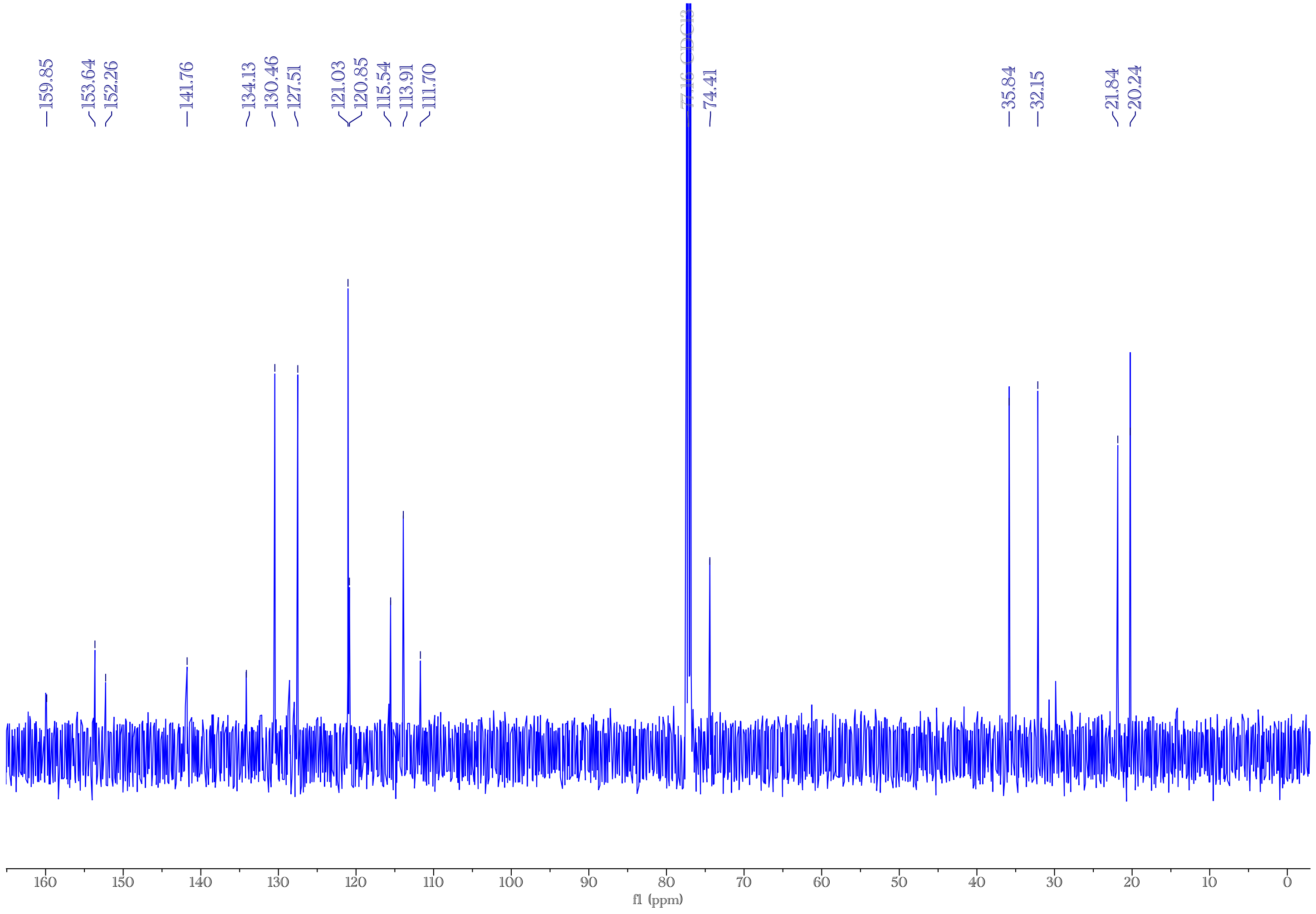

Figure S15. HSQC of radulanin L (**4**) in $CDCl_3$

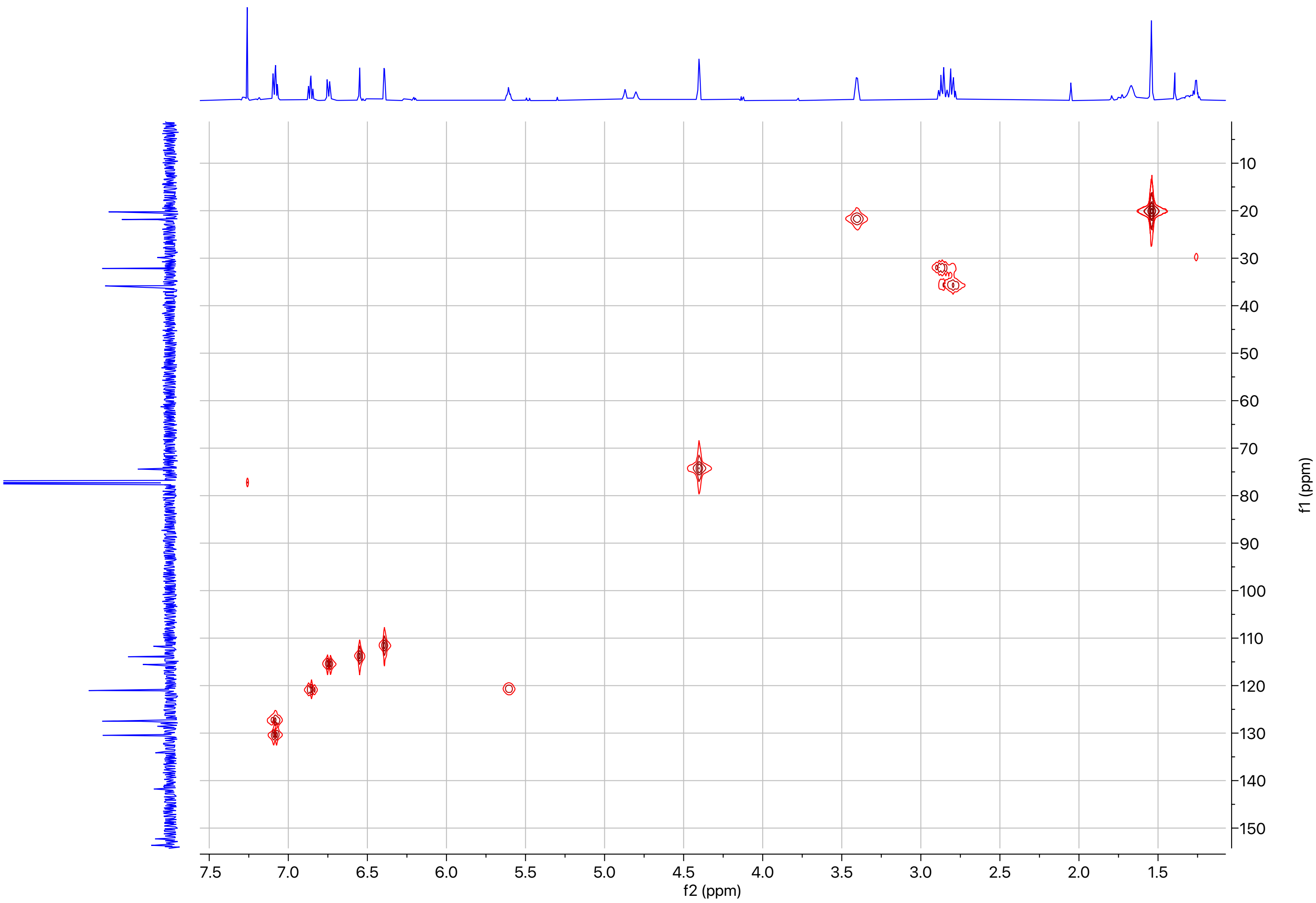

Figure S16. HMBC of radulanin L (**4**) in $CDCl_3$

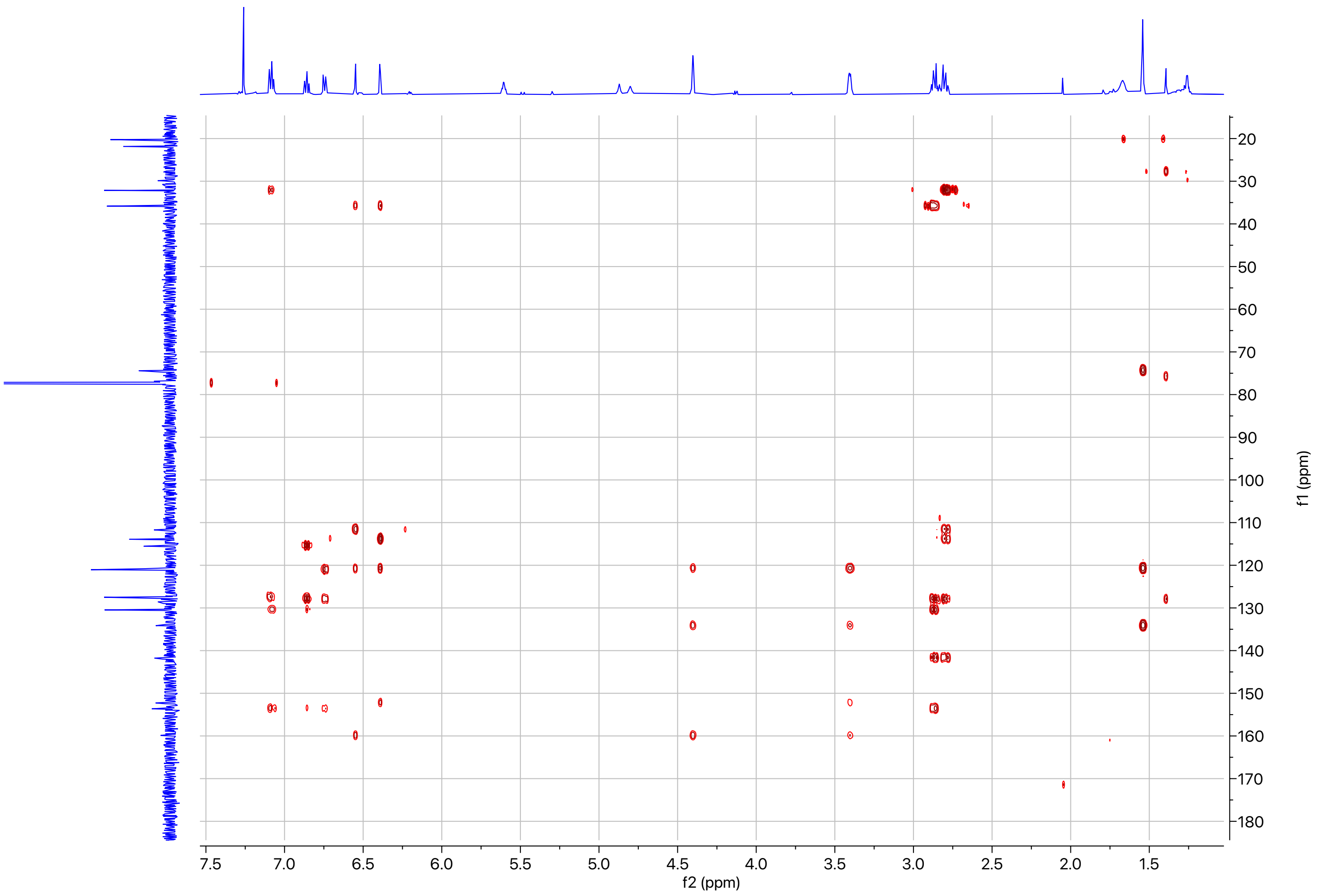

Molecule 1

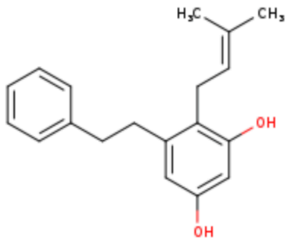

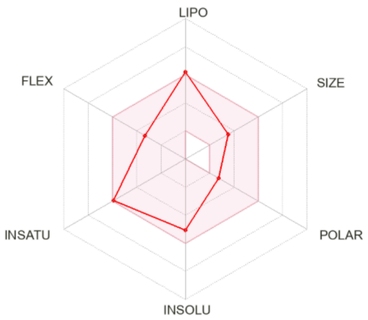

SMILES CC(=CCc1c(CCc2ccccc2)cc(cc1O)O)C

Physicochemical Properties	
Formula	C19H22O2
Molecular weight	282.38 g/mol
Num. heavy atoms	21
Num. arom. heavy atoms	12
Fraction Csp3	0.26
Num. rotatable bonds	5
Num. H-bond acceptors	2
Num. H-bond donors	2
Molar Refractivity	88.47
TPSA	40.46 Å²

Lipophilicity	
Log $P_{o/w}$ (iLOGP)	3.04
Log $P_{o/w}$ (XLOGP3)	5.35
Log $P_{o/w}$ (WLOGP)	4.39
Log $P_{o/w}$ (MLOGP)	4.09
Log $P_{o/w}$ (SILICOS-IT)	4.93
Consensus Log $P_{o/w}$	4.36

Water Solubility	
Log *S* (ESOL)	-5.05
Solubility	2.49e-03 mg/ml ; 8.83e-06 mol/l
Class	Moderately soluble
Log *S* (Ali)	-5.95
Solubility	3.15e-04 mg/ml ; 1.12e-06 mol/l
Class	Moderately soluble
Log *S* (SILICOS-IT)	-5.84
Solubility	4.09e-04 mg/ml ; 1.45e-06 mol/l
Class	Moderately soluble

Pharmacokinetics	
GI absorption	High
BBB permeant	Yes
P-gp substrate	No
CYP1A2 inhibitor	Yes
CYP2C19 inhibitor	Yes
CYP2C9 inhibitor	No
CYP2D6 inhibitor	Yes
CYP3A4 inhibitor	Yes
Log K_p (skin permeation)	-4.22 cm/s

Druglikeness	
Lipinski	Yes; 0 violation
Ghose	Yes
Veber	Yes
Egan	Yes
Muegge	No; 1 violation: XLOGP3>5
Bioavailability Score	0.55

Medicinal Chemistry	
PAINS	0 alert
Brenk	1 alert: isolated_alkene
Leadlikeness	No; 1 violation: XLOGP3>3.5
Synthetic accessibility	2.81

SwissADME: a free web tool to evaluate pharmacokinetics, drug-likeness and medicinal chemistry friendliness of small molecules. *Sci. Rep.* (2017) 7:42717.

Molecule 2

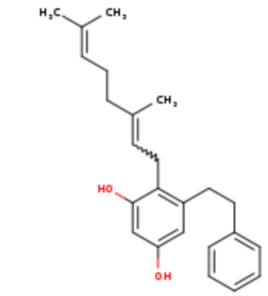

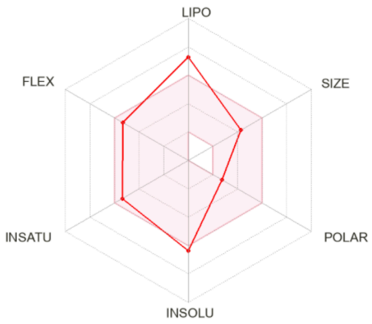

SMILES C/C(=C\Cc1c(O)cc(cc1CCc1ccccc1)O)/CCC=C(C)C

Physicochemical Properties	
Formula	C24H30O2
Molecular weight	350.49 g/mol
Num. heavy atoms	26
Num. arom. heavy atoms	12
Fraction Csp3	0.33
Num. rotatable bonds	8
Num. H-bond acceptors	2
Num. H-bond donors	2
Molar Refractivity	112.03
TPSA	40.46 Å²

Lipophilicity	
Log $P_{o/w}$ (iLOGP)	4.04
Log $P_{o/w}$ (XLOGP3)	7.21
Log $P_{o/w}$ (WLOGP)	6.12
Log $P_{o/w}$ (MLOGP)	5.11
Log $P_{o/w}$ (SILICOS-IT)	6.61
Consensus Log $P_{o/w}$	5.82

Water Solubility	
Log *S* (ESOL)	-6.37
Solubility	1.50e-04 mg/ml ; 4.28e-07 mol/l
Class	Poorly soluble
Log *S* (Ali)	-7.88
Solubility	4.59e-06 mg/ml ; 1.31e-08 mol/l
Class	Poorly soluble
Log *S* (SILICOS-IT)	-7.09
Solubility	2.85e-05 mg/ml ; 8.13e-08 mol/l
Class	Poorly soluble

Pharmacokinetics	
GI absorption	High
BBB permeant	No
P-gp substrate	Yes
CYP1A2 inhibitor	Yes
CYP2C19 inhibitor	Yes
CYP2C9 inhibitor	No
CYP2D6 inhibitor	Yes
CYP3A4 inhibitor	Yes
Log K_p (skin permeation)	-3.32 cm/s

Druglikeness	
Lipinski	Yes; 1 violation: MLOGP>4.15
Ghose	No; 1 violation: WLOGP>5.6
Veber	Yes
Egan	No; 1 violation: WLOGP>5.88
Muegge	No; 1 violation: XLOGP3>5
Bioavailability Score	0.55

Medicinal Chemistry	
PAINS	0 alert
Brenk	1 alert: isolated_alkene
Leadlikeness	No; 3 violations: MW>350, Rotors>7, XLOGP3>3.5
Synthetic accessibility	3.41

SwissADME: a free web tool to evaluate pharmacokinetics, drug-likeness and medicinal chemistry friendliness of small molecules. *Sci. Rep.* (2017) 7:42717.

Molecule 3

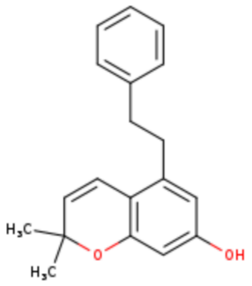

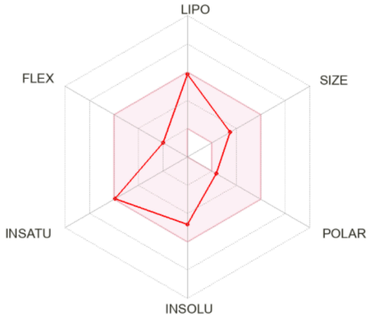

SMILES Oc1cc(CCc2ccccc2)c2c(c1)OC(C=C2)(C)C

Physicochemical Properties	
Formula	C19H20O2
Molecular weight	280.36 g/mol
Num. heavy atoms	21
Num. arom. heavy atoms	12
Fraction Csp3	0.26
Num. rotatable bonds	3
Num. H-bond acceptors	2
Num. H-bond donors	1
Molar Refractivity	86.85
TPSA	29.46 Å²

Lipophilicity	
Log $P_{o/w}$ (iLOGP)	3.19
Log $P_{o/w}$ (XLOGP3)	4.71
Log $P_{o/w}$ (WLOGP)	4.25
Log $P_{o/w}$ (MLOGP)	3.70
Log $P_{o/w}$ (SILICOS-IT)	4.68
Consensus Log $P_{o/w}$	4.11

Water Solubility	
Log *S* (ESOL)	-4.77
Solubility	4.76e-03 mg/ml ; 1.70e-05 mol/l
Class	Moderately soluble
Log *S* (Ali)	-5.06
Solubility	2.46e-03 mg/ml ; 8.76e-06 mol/l
Class	Moderately soluble
Log *S* (SILICOS-IT)	-6.01
Solubility	2.73e-04 mg/ml ; 9.75e-07 mol/l
Class	Poorly soluble

Pharmacokinetics	
GI absorption	High
BBB permeant	Yes
P-gp substrate	No
CYP1A2 inhibitor	Yes
CYP2C19 inhibitor	Yes
CYP2C9 inhibitor	No
CYP2D6 inhibitor	Yes
CYP3A4 inhibitor	No
Log K_p (skin permeation)	-4.67 cm/s

Druglikeness	
Lipinski	Yes; 0 violation
Ghose	Yes
Veber	Yes
Egan	Yes
Muegge	Yes
Bioavailability Score	0.55

Medicinal Chemistry	
PAINS	0 alert
Brenk	0 alert
Leadlikeness	No; 1 violation: XLOGP3>3.5
Synthetic accessibility	3.43

SwissADME: a free web tool to evaluate pharmacokinetics, drug-likeness and medicinal chemistry friendliness of small molecules. *Sci. Rep.* (2017) 7:42717.

Molecule 4

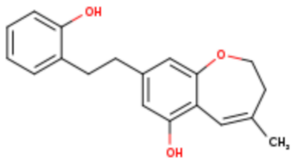

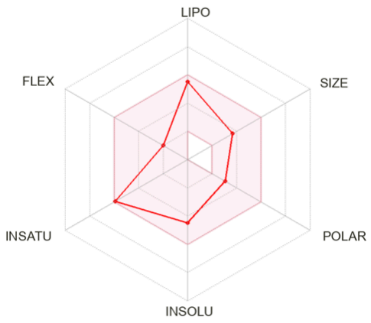

SMILES CC1=Cc2c(O)cc(cc2OCC1)CCc1ccccc1O

Physicochemical Properties	
Formula	C19H20O3
Molecular weight	296.36 g/mol
Num. heavy atoms	22
Num. arom. heavy atoms	12
Fraction Csp3	0.26
Num. rotatable bonds	3
Num. H-bond acceptors	3
Num. H-bond donors	2
Molar Refractivity	88.83
TPSA	49.69 Å²

Lipophilicity	
Log $P_{o/w}$ (iLOGP)	2.78
Log $P_{o/w}$ (XLOGP3)	4.14
Log $P_{o/w}$ (WLOGP)	3.96
Log $P_{o/w}$ (MLOGP)	3.08
Log $P_{o/w}$ (SILICOS-IT)	4.34
Consensus Log $P_{o/w}$	3.66

Water Solubility	
Log *S* (ESOL)	-4.49
Solubility	9.56e-03 mg/ml ; 3.23e-05 mol/l
Class	Moderately soluble
Log *S* (Ali)	-4.89
Solubility	3.81e-03 mg/ml ; 1.29e-05 mol/l
Class	Moderately soluble
Log *S* (SILICOS-IT)	-5.55
Solubility	8.31e-04 mg/ml ; 2.81e-06 mol/l
Class	Moderately soluble

Pharmacokinetics	
GI absorption	High
BBB permeant	Yes
P-gp substrate	No
CYP1A2 inhibitor	Yes
CYP2C19 inhibitor	Yes
CYP2C9 inhibitor	No
CYP2D6 inhibitor	Yes
CYP3A4 inhibitor	No
Log K_p (skin permeation)	-5.17 cm/s

Druglikeness	
Lipinski	Yes; 0 violation
Ghose	Yes
Veber	Yes
Egan	Yes
Muegge	Yes
Bioavailability Score	0.55

Medicinal Chemistry	
PAINS	0 alert
Brenk	0 alert
Leadlikeness	No; 1 violation: XLOGP3>3.5
Synthetic accessibility	3.61

SwissADME: a free web tool to evaluate pharmacokinetics, drug-likeness and medicinal chemistry friendliness of small molecules. *Sci. Rep.* (2017) 7:42717.

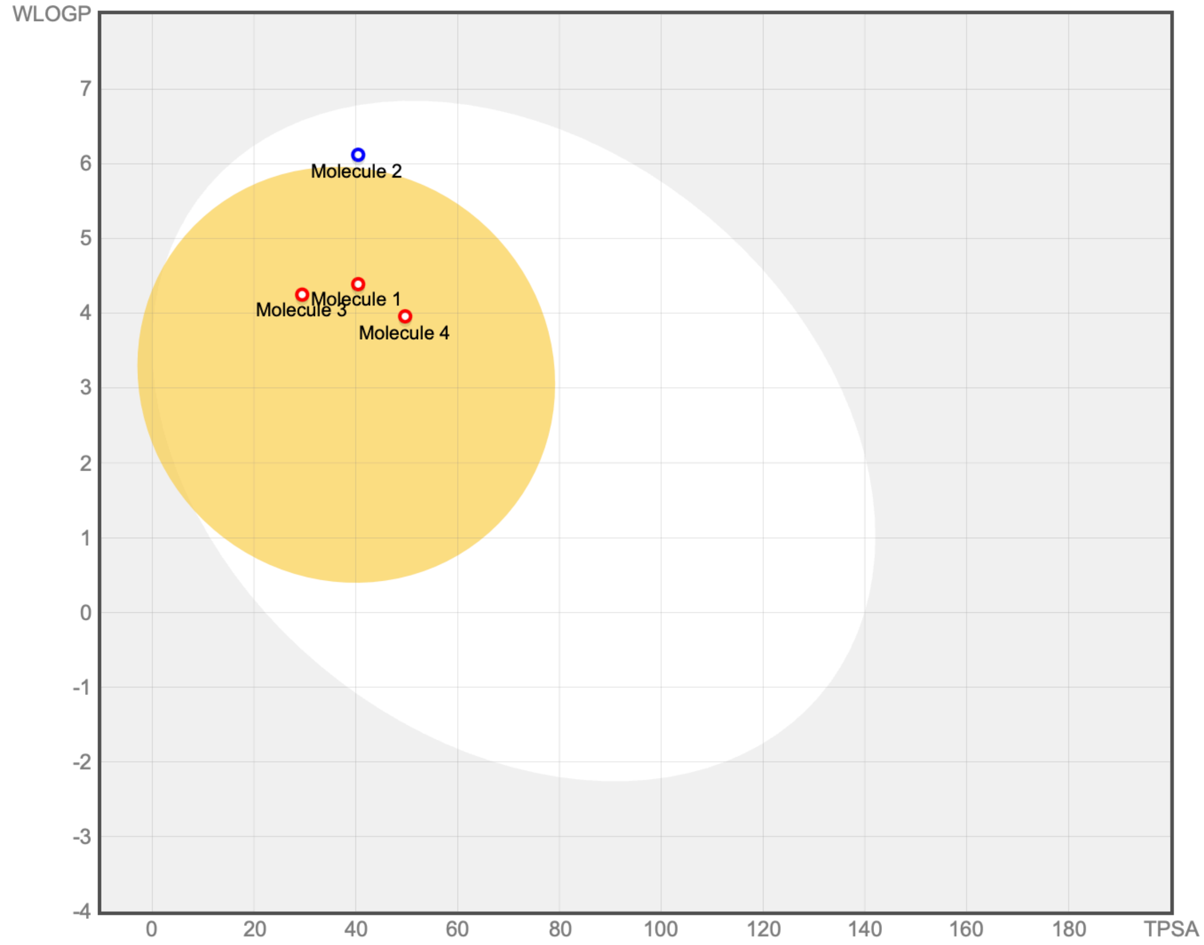

BOILED-Egg Model that shows the lipophilicity and polarity prediction of compounds **1**-**4.** Points inside the white ellipse indicate compounds likely to be absorbed in the gastrointestinal tract. Points inside the yellow ellipse indicate compounds likely to cross the BBB and reach the CNS. Blue points represent predicted P-glycoprotein substrates (PGP+), and red points represent predicted non-substrates (PGP−) [Reference: A BOILED-Egg To Predict Gastrointestinal Absorption and Brain Penetration of Small Molecules. A. Daina, V. Zoete, *ChemMedChem* **2016**, *11*, 1117].
